# Mastering the canvas of life: Identifying the antecedents of sense of control using a lagged exposure‐wide approach

**DOI:** 10.1111/aphw.12618

**Published:** 2024-12-12

**Authors:** Eric S. Kim, Ying Chen, Joanna H. Hong, Margie E. Lachman, Tyler J. VanderWeele

**Affiliations:** ^1^ Department of Psychology University of British Columbia Canada; ^2^ Human Flourishing Program, Institute for Quantitative Social Science Harvard University Cambridge MA USA; ^3^ Lee Kum Sheung Center for Health and Happiness Harvard T.H. Chan School of Public Health Boston MA USA; ^4^ Department of Epidemiology Harvard T.H. Chan School of Public Health Boston MA USA; ^5^ Department of Psychology Brandeis University Boston MA USA; ^6^ Department of Biostatistics Harvard T.H. Chan School of Public Health Boston MA USA

**Keywords:** health and well‐being, mastery, older adults, outcome‐wide epidemiology, sense of control

## Abstract

Accumulating studies have documented strong associations between a higher sense of control and improved health and well‐being outcomes. However, less is known about the determinants of increased sense of control. Our analysis used data from 13,771 older adults in the Health and Retirement Study (HRS)—a diverse, longitudinal, and national study of adults aged >50 in the United States. Using generalized linear regression models, with a lagged exposure‐wide approach, we evaluated how *changes* in 59 predictors (i.e., physical health, health behavior, and psychosocial factors) over a 4‐year period (between t_0_;2006/2008 and t_1_;2010/2012) might lead to changes in sense of control another 4‐years later (t_2_;2014/2016). After adjusting for a rich set of baseline covariates, changes in some health behaviors (e.g., sleep problems), physical health conditions (e.g., physical functioning limitations, eyesight), and psychosocial factors (e.g., positive affect, purpose in life) were associated with changes in sense of control four years later. However, there was little evidence that other factors were associated with a subsequent sense of control. A key challenge in advancing intervention development is the identification of antecedents that predict a sense of control. Our results identified several novel targets for interventions and policies aimed at increasing a sense of control.

## INTRODUCTION

Accumulating evidence shows that a higher sense of control—the perception that one has the ability to influence one's environment and elicit desired outcomes—(Bandura, 1997; Lachman et al., 2011) is a promising *health asset*, as it has been associated with reduced risk of chronic conditions (e.g., lung disease, cognitive impairment, stroke, cardiovascular disease) (Hong et al., 2021; Infurna et al., 2018; Robinson & Lachman, 2018) and mortality among older adults (Hong et al., 2021; Infurna et al., 2011; Kubzansky et al., 2018). Several hypothesized mechanisms underlie these associations, including the cultivation of enhanced psychological well‐being (e.g., purpose in life, positive affect, optimism) (Elliot et al., 2018; Hong et al., 2021), decreased psychological distress (e.g., negative affect, depressive symptoms, and anxiety) (Elliot et al., 2018; Hong et al., 2021), and better health behaviors (e.g., increased: physical activity, decreased: sleep problems) (Hong et al., 2021; Robinson & Lachman, 2018).

Sense of control has been described in numerous ways and has many intellectual cousins (e.g., control beliefs, self‐efficacy, locus of control, perceived control, learned helplessness), but we use a widely accepted conceptual framework that combines two subcomponents: personal mastery and perceived constraints (Lachman et al., 2011; Rodin, 1990) and refers to this composite construct as *sense of control* (Robinson & Lachman, 2017). This highlights the need for strategies that enhance the sense of control. Sense of control is influenced by social‐structural factors, genetics, and changing life circumstances (Lachman & Weaver, 1998; Waaktaar & Torgersen, 2013). However, research has shown that it is modifiable through interventions (e.g., instilling beliefs that a sense of control is malleable, a cognitive behavioral therapy that focuses on restructuring maladaptive thoughts around a sense of control, increasing autonomy (e.g., how and when leisure‐activities or work tasks are performed)) (Lachman et al., 2011; Lachman et al., 2015; Robinson & Lachman, 2017). One key factor hindering further intervention development is the identification of antecedents that predict a sense of control.

While some literature has identified sociodemographic factors that predict subsequent sense of control (e.g., gender, age, education) (Drewelies et al., 2017; Infurna et al., 2011; Lachman & Weaver, 1998; Mirowsky, 1995), accumulating evidence shows that a higher sense of control is predicted by other potentially modifiable factors, including: social factors (e.g., increased: social participation, volunteering, social network contact, perceived support from network members; lower: loneliness) (Curtis et al., 2018; Drewelies et al., 2017; Gerstorf et al., 2011; Infurna et al., 2011; Infurna & Okun, 2015; Krause, 1987; Lang et al., 1997; McAvay et al., 1996), psychological factors (e.g., decreased: depression and negative affect) (Infurna & Okun, 2015; McAvay et al., 1996; Schulz & Heckhausen, 1998), cognitive abilities (Lachman et al., 2009), and physical health factors (increased: self rated health; decreased: physical functioning limitations, chronic illnesses) (Cairney et al., 2007; Drewelies et al., 2017; Infurna & Okun, 2015).

These prior studies have made seminal contributions to the literature, but remain limited in some ways. First, while an increasing number of studies use longitudinal designs, many older studies are cross‐sectional and cannot assess directionality. Second, many studies did not adequately adjust for key potential confounders (e.g., only adjusting for basic demographics). Third, many studies use data from small samples or specific subpopulations (e.g., people with spinal cord injury) (Li et al., 2021), limiting generalizability to broader populations. Fourth, most studies only evaluated a limited number of predictors, so that we cannot directly compare effect sizes, which is helpful when trying to determine intervention targets that might produce the largest effects. Fifth, many candidate antecedents of sense of control have been understudied or not at all in older adults. Sixth, existing longitudinal studies evaluated predictors of sense of control accumulated across the life‐course, rather than changes in predictors. Adjusting for pre‐baseline predictors and outcomes allows researchers to ask a slightly different question more aligned with the interests of interventionists and policy‐makers: if a given predictor is modified, what change in sense of control might we observe?

In our study, we used a *lagged exposure‐wide* analytic approach (see *Statistical Analysis* section) (VanderWeele et al., 2020), to evaluate how changes in 59 predictors (i.e., physical health, health behaviors, psychosocial factors) over a 4‐year period might lead to changes in sense of control another 4‐years later. This hypothesis‐generating, data‐driven approach allowed us to identify promising antecedents of sense of control, which can then undergo further investigation in future studies. We chose these 59 predictors because they are frequently included in the conceptualization of key gerontological models that characterize the antecedents, processes, and outcomes that foster people's ability to age well (Aldwin & Igarashi, 2015; Depp & Jeste, 2006; Reich et al., 2010; Rowe & Kahn, 1987, 2015; Ryff & Singer, 2009). Further, many of the candidate predictors are modifiable, or likely modifiable with further research. This approach helped us assess a broad spectrum of potential antecedents of sense of control and compare effect sizes as we used the same: population, study design, analytic methods, and covariates.

## METHODS

### Study population

We used data from the Health and Retirement Study (HRS), which is a national representative panel study of adults in the United States aged >50. HRS first assessed psychosocial data in 2006. In this year, a random 50% of participants were selected to complete an enhanced face‐to‐face (EFTF) interview. The other 50% were assessed in the next wave (2008). After completing the interview, participants completed a psychosocial questionnaire and then mailed it back to the University of Michigan (response rates: 88% in 2006 and 84% in 2008) (Smith et al., 2017). Each participant completes this psychosocial questionnaire every four years. Data from the 2006 and 2008 sub‐cohorts were combined to increase sample size and statistical power. Participants were excluded if they did not report psychosocial data in this pre‐baseline wave (since over half of the study predictors were psychosocial factors) resulting in a final sample of 13,771 participants.

We used data from three‐time points, each spaced four years apart: 1) covariates were assessed in the pre‐baseline wave (t_0_;2006/2008), 2) candidate predictors were assessed in the baseline wave (t_1_;2010/2012), and 3) our outcome (sense of control) was assessed in the outcome wave (t_2_;2014/2016). The HRS is sponsored by the National Institute on Aging (NIA U01AG009740) and conducted by the University of Michigan (http://hrsonline.isr.umich.edu/) (Sonnega et al., 2014). The ethics board at the University of British Columbia exempted our study from review because it used de‐identified and publicly available data.

### Measures

#### Sense of control

Sense of control was assessed by averaging two subscales: sense of personal mastery and perceived constraints (Lachman & Weaver, 1998). Using a 6‐point Likert‐scale (1 = Strongly disagree; 6 = Strongly agree), participants indicated the extent to which they agreed with five items measuring personal mastery (e.g., “I can do just about anything I really set my mind to”) and five items measuring perceived constraints (e.g. “I often feel helpless in dealing with the problems of life”). Items on the constraints subscale were reverse‐coded and the mean of all 10 items on both subscales was averaged together to create a composite scale, where higher scores indicate a higher sense of control (*α* = 0.88).

#### Covariates

We adjusted for a substantial number of covariates in the pre‐baseline wave (t_0_;2006/2008), including sociodemographics (age (continuous), gender (male/female), race/ethnicity (White, African‐American, Hispanic, Other), marital status (married/not married), income (<$50,000, $50,000–$74,999, $75,000–$99,999, ≥$100,000), total wealth (based on quintiles of the score distribution for total wealth in this sample), educational attainment (no degree, GED/high school diploma, ≥college degree), health insurance (yes/no), geographic region (Northeast, Midwest, South, West), personality (openness, conscientiousness, extraversion, agreeableness, neuroticism; continuous), and childhood abuse (yes/no)). We also adjusted for all 59 candidate predictors, and the pre‐baseline value of sense of control, simultaneously in the pre‐baseline wave.

#### Predictors

We evaluated 59 candidate predictors in the baseline wave (t_1_;2010/2012) including measures of: (1) health behaviors (physical activity, smoking, heavy drinking, sleep problems); (2) physical health (number of chronic conditions, diabetes, hypertension, stroke, cancer, heart disease, lung disease, arthritis, overweight/obesity, physical functioning limitations, cognitive impairment, chronic pain, self‐rated health, hearing, eyesight); (3) psychological well‐being (positive affect, life satisfaction, optimism, purpose in life, health mastery, financial mastery); (4) psychological distress (depression, depressive symptoms, hopelessness, negative affect, anxiety, trait anger, state anger, cynical hostility, stressful life events, financial strain, daily discrimination, major discrimination); (5) social factors (loneliness, living with spouse, frequency of contact in three separate relationship categories: (i) children, (ii) other family, and (iii) friends, closeness with spouse, number of close (i) children, (ii) other family, and (iii) friends, positive social support from (i) spouse, (ii) children, (iii) other family, and (iv) friends, negative social strain from (i) spouse, (ii) children, (iii) other family, and (iv) friends, volunteer activity, helping friends, neighbors, and relatives, religious service attendance, social status ladder ranking, and change in social status ladder ranking); and (6) in the labor force. HRS Materials and Appendix Text #1 provide further details about each variable (Fisher et al., 2005; Jenkins et al., 2008; Smith et al., 2017).

We evaluated health mastery and financial mastery independently as predictors, as these are distinct areas specifically related to health and financial management. This distinction allowed us to investigate if these particular areas independently contribute to a broader sense of control.

#### Multiple imputation

All missing exposures, covariates, and outcome variables were imputed using multiple imputations by chained equations, and five datasets were created. We chose this approach because it provides a potentially more accurate approach than other methods of handling missing data (Groenwold et al., 2012; Moons et al., 2006; Sterne et al., 2009) and helps address problems that emerge due to attrition (Asendorpf et al., 2014; Cumming & Goldstein, 2016; Harel et al., 2018; Rawlings et al., 2017; van Ginkel et al., 2019; Weuve et al., 2015).

### Statistical analysis

We used a lagged exposure‐wide approach (VanderWeele et al., 2020) and ran separate models for each exposure (see Appendix Text #2 for further details). In our primary analyses, sense of control was a continuous outcome (standardized at mean = 0 and standard deviation = 1). Further, continuous predictors were standardized so their effect sizes could be interpreted as a standard deviation change in the exposure.

We used linear regression models and individually regressed sense of control at the outcome wave (t_2_:2014/2016) on each baseline candidate predictor (at t_1_;2010/2012) in separate models. In these analyses, we controlled for all covariates in the previous wave (t_0_;2006/2008). To assess change in each predictor, we adjusted for prior values of all predictors. Additionally, to minimize the possibility of reverse causation, we also adjusted for pre‐baseline sense of control. Thus, for exposures, the effect estimate corresponds to associations between the exposure at baseline (at t_1_;2010/2012) and sense of control at the outcome wave (t_2_:2014/2016), conditional on the exposure, outcome, and covariates in the pre‐baseline wave (at t_0_;2006/2008). Because multiple testing practices vary widely and are continuously evolving (Dunn, 1961; VanderWeele & Mathur, 2019), we marked multiple p‐value cutoffs, including Bonferroni‐corrected ones, and provided confidence intervals.

#### Additional analyses

We conducted three additional analyses. First, to evaluate the robustness of our results to potential unmeasured confounding, we calculated E‐values. This method allows researchers to assess the minimum strength of unmeasured confounding on the risk ratio scale (with both the exposure and the outcome) needed to explain away the observed association between the exposure and outcome (VanderWeele & Ding, 2017). A high E‐value signifies that any unmeasured confounder would need to have a strong association with both the treatment and the outcome to explain away the observed effect. This suggests that the results are more likely to reflect a true causal relationship. A low E‐value signifies the opposite. Second, we conducted additional analyses that separately evaluated mastery and perceived constraints as separate outcomes. We conducted these analyses because this approach would allow us to evaluate the unique contribution of each predictor (i.e., physical health, health behavior, and psychosocial factors), and potentially varying effect sizes on the outcome variables – the two separate facets of control beliefs. Third, we conducted sensitivity analyses that did not standardize any of the variables.

## RESULTS

In the pre‐baseline wave (t_0_;2006/2008), when all covariates were assessed, the average age of participants was 69 years old (SD = 10), more likely women (58%) and married (62%). Table 1 summarizes participant characteristics. Table A1 describes the changes in sense of control from the pre‐baseline wave (t0) to the outcome wave (t2). We observed that the mean levels of control beliefs remained consistent across waves t_0_ to t_2_, indicating no substantial mean level change within the population. However, stability correlations of 0.41 between t_0_ and t_1_, and 0.32 between t_1_ and t_2_, highlight individual differences in rank order changes, suggesting that while control beliefs on average remained stable, individuals varied in their experiences of sense of control, with some reporting increases and others decreases (Table A1). Table 2 shows the associations between the candidate predictors and the subsequent sense of control. When considering health behaviors, 1 out of 4 predictors were associated with a subsequent sense of control. Worse sleep was associated with a lower subsequent sense of control (*β* = −0.05, 95% confidence interval [CI] = −0.10, −0.01). For physical health indicators, 2 out of 15 candidate predictors were associated with a subsequent sense of control. Specifically, better self‐rated health (*β* = 0.08, 95% CI = 0.03, 0.12) and fewer physical functioning limitations (*β* = −0.09, 95% CI = −0.16, −0.01), were each associated with a higher subsequent sense of control. Additionally, we also compared those who were lost to follow‐up versus (vs.) those who stayed in HRS (Table A2). Compared to those who were lost to follow‐up, people who stayed in HRS were generally healthier, wealthier, more highly educated, and had better health behaviors and mental health.

**TABLE 1:** Characteristics of participants at pre‐baseline (N = 13,605)^a^ ^,^ ^b^ ^,^ ^c^.

Participant characteristics	No. (%)	Mean (SD)
***Sociodemographic factors***		
Age (yr; range: 52–104)		69.24 (9.63)
Female (%)	8,041 (58.39)	
Race/ethnicity (%)		
White	10,642 (77.28)	
Black	1,761 (12.79)	
Hispanic	1,088 (7.90)	
Other	279 (2.03)	
Married (%)	8,589 (62.37)	
Annual household income (%)		
<$50,000	8,362 (60.72)	
$50,000–$74,999	2,127 (15.45)	
$75,000–$99,999	1,176 (8.54)	
≥$100,000	2,106 (15.29)	
Total wealth (%)		
1st quintile	2,770 (20.11)	
2nd quintile	2,744 (19.93)	
3rd quintile	2,749 (19.96)	
4th quintile	2,757 (20.02)	
5th quintile	2,751 (19.98)	
Education (%)		
<high school	2,715 (19.76)	
High school	7,513 (54.67)	
≥college	3,515 (25.58)	
Health insurance (%)	13,183 (95.79)	
Geographic region (%)		
Northeast	2,091 (15.21)	
Midwest	3,594 (26.14)	
South	5,497 (39.98)	
West	2,566 (18.66)	
Personality factors		
Openness (range: 1–4)		2.93 (0.44)
Conscientiousness (range: 1–4)		3.35 (0.48)
Extraversion (range: 1–4)		3.20 (0.55)
Agreeableness (range: 1–4)		3.53 (0.47)
Neuroticism (range: 1–4)		2.04 (0.61)
Childhood abuse (%)	849 (6.28)	
***Health behaviors***		
Frequent physical activity (%)	9,869 (71.74)	
Smoking (%)	1,725 (12.61)	
Heavy drinking (%)	791 (6.98)	
Sleep problems (%)	3,055 (42.08)	
***Physical health***		
Number of physical conditions (range: 0–8)		2.63 (1.46)
Diabetes (%)	2,728 (19.81)	
Hypertension (%)	7,847 (56.98)	
Stroke (%)	1,107 (8.04)	
Cancer (%)	2,077 (15.08)	
Heart disease (%)	3,354 (24.36)	
Lung disease (%)	1,298 (9.43)	
Arthritis (%)	8,291 (60.21)	
Overweight/obese (%)	9,491 (69.75)	
Physical functioning limitations (%)	3,329 (24.17)	
Cognitive impairment (%)	2,705 (19.96)	
Chronic pain (%)	4,754 (34.53)	
Self‐rated health (range: 1–5)		3.15 (1.09)
Hearing (range: 1–5)		3.32 (1.10)
Eyesight (range: 1–6)		4.18 (1.00)
***Psychological well‐being***		
Positive affect (range: 1–5)		3.58 (0.74)
Life satisfaction (range: 1–7)		5.03 (1.47)
Optimism (range: 1–6)		4.46 (0.96)
Purpose in life (range: 1–6)		4.58 (0.93)
Mastery (range: 1–6)		4.75 (1.11)
Health mastery (range: 0–10)		7.23 (2.39)
Financial mastery (range: 0–10)		7.34 (2.65)
***Psychological distress***		
Depression (%)	1,881 (13.88)	
Depressive symptoms (range: 0–8)		1.40 (1.93)
Hopelessness (range: 1–6)		2.40 (1.29)
Negative affect (range: 1–5)		1.68 (0.64)
Perceived constraints (range: 1–6)		2.23 (1.20)
Anxiety (range: 1–4)		1.57 (0.59)
Trait anger (range: 1–4)		2.17 (0.68)
State anger (range: 1–4)		1.50 (0.51)
Cynical hostility (range: 1–6)		2.96 (1.14)
Stressful life events (range: 0–5)		0.23 (0.55)
Financial strain (range: 1–5)		1.96 (1.00)
Daily discrimination (range: 1–6)		1.62 (0.74)
Major discrimination (range: 0–6)		0.46 (0.88)
***Social factors***		
Loneliness (range: 1–3)		1.48 (0.54)
Living with spouse/partner (%)	8,796 (65.74)	
Contact children (%)		
<every few months	1,845 (13.76)	
1‐2x/month	1,510 (11.26)	
1‐2x/week	4,134 (30.83)	
≥3x/week	5,919 (44.15)	
Contact other family (%)		
<every few months	3,274 (24.37)	
1‐2x/month	3,124 (23.25)	
1‐2x/week	3,679 (27.38)	
≥3x/week	3,358 (24.99)	
Contact friends (%)		
<every few months	2,268 (16.79)	
1‐2x/month	2,482 (18.37)	
1‐2x/week	4,810 (35.60)	
≥3x/week	3,951 (29.24)	
Closeness with spouse (range: 1–4)		3.47 (0.73)
Number of close children		2.81 (3.73)
Number of close other family		3.87 (5.56)
Number of close friends		4.54 (6.03)
Positive social support from spouse (range: 1–4)		3.46 (0.66)
Positive social support from children (range: 1–4)		3.27 (0.72)
Positive social support from other family (range: 1–4)		2.89 (0.87)
Positive social support from friends (range: 1–4)		3.05 (0.75)
Social strain from spouse (range: 1–4)		1.98 (0.68)
Social strain from children (range: 1–4)		1.70 (0.64)
Social strain from other family (range: 1–4)		1.57 (0.62)
Social strain from friends (range: 1–4)		1.84 (0.43)
Volunteering (%)		
0 h/year	8,928 (64.91)	
1–49 h/year	1,530 (11.12)	
50–99 h/year	1,085 (7.89)	
100–199 h/year	1,191 (8.66)	
≥200 h/year	1,021 (7.42)	
Helping friends/neighbors/relatives (%)		
0 h/year	6,626 (48.26)	
1–49 h/year	3,218 (23.43)	
50–99 h/year	1,836 (13.37)	
100–199 h/year	1,190 (8.67)	
≥200 h/year	859 (6.26)	
Religious service attendance (%)		
Not at all	3,455 (25.11)	
<1x/week	4,300 (31.25)	
≥1x/week	6,007 (43.65)	
Social status ladder (range: 1–10)		6.5 (1.8)
Change in social status ladder (%)		
Moved down	1,268 (9.58)	
No change	10,295 (77.79)	
Moved up	1,671 (12.63)	
In labor force (%)	4,782 (34.73)	

^a^ This table was created based on non‐imputed data.

^b^ All variables in Table 1 were used as covariates, and assessed in the pre‐baseline wave (t_0_;2006/2008).

^c^ The percentages in some sections may not add up to 100% due to rounding.

**TABLE 2:** Candidate predictors of sense of control (Health and Retirement Study [HRS]: *N* = 13,771).

Candidate predictor	Beta	95% CI
Health behaviors		
Frequent physical activity	0.01	−0.05, 0.07
Smoking	−0.04	−0.18, 0.09
Heavy drinking	0.01	−0.10, 0.12
Sleep problems	−0.05	−0.10, −0.01*
Physical health		
Number of physical conditions	−0.04	−0.10, 0.02
Diabetes	−0.04	−0.13, 0.05
Hypertension	0.00	−0.09, 0.08
Stroke	−0.04	−0.13, 0.05
Cancer	0.03	−0.06, 0.11
Heart disease	0.00	−0.09, 0.08
Lung disease	0.02	−0.08, 0.13
Arthritis	−0.07	−0.18, 0.05
Overweight/obese	−0.07	−0.14, 0.00
Physical functioning limitations	−0.09	−0.16, −0.01*
Cognitive impairment	−0.03	−0.10, 0.05
Chronic pain	−0.05	−0.11, 0.01
Self‐rated health	0.08	0.03, 0.12**
Hearing	0.01	−0.03, 0.04
Eyesight	0.04	0.00, 0.07*
Psychological well‐being		
Positive affect	0.15	0.11, 0.18***
Life satisfaction	0.10	0.07, 0.14***
Optimism	0.13	0.10, 0.16***
Purpose in life	0.14	0.08, 0.19***
Health mastery	0.10	0.07, 0.12***
Financial mastery	0.10	0.07, 0.13***
Psychological distress		
Depression	−0.10	−0.20, 0.00
Depressive symptoms	−0.05	−0.09, −0.02**
Hopelessness	−0.14	−0.18, −0.11***
Negative affect	−0.11	−0.13, −0.09***
Anxiety	−0.10	−0.13, −0.07***
Trait anger	−0.05	−0.07, −0.02**
State anger	−0.03	−0.06, −0.01**
Cynical hostility	−0.06	−0.08, −0.03***
Stressful life events	−0.02	−0.05, 0.01
Financial strain	−0.05	−0.08, −0.01*
Daily discrimination	−0.06	−0.09, −0.04***
Major discrimination	−0.01	−0.07, 0.04
Social factors		
Loneliness	−0.08	−0.10, −0.05***
Living with spouse	−0.08	−0.17, 0.00
Contact children		
<Every few months	Reference	Reference
1–2×/month	0.06	−0.05, 0.17
1–2×/week	0.07	−0.03, 0.16
>3×/week	0.09	−0.01, 0.19
Contact other family		
<Every few months	Reference	Reference
1–2×/month	0.03	−0.02, 0.09
1–2×/week	0.02	−0.04, 0.08
>3×/week	0.02	−0.06, 0.10
Contact friends		
<Every few months	Reference	Reference
1–2×/month	0.05	−0.02, 0.13
1–2×/week	0.06	−0.02, 0.14
>3×/week	0.09	0.01, 0.16*
Closeness with spouse	−0.01	−0.08, 0.05
Number of close children	0.01	−0.02, 0.04
Number of close other family	0.01	−0.02, 0.04
Number of close friends	0.02	−0.02, 0.06
Positive social support from spouse	0.04	−0.01, 0.09
Positive social support from children	0.06	0.03, 0.10**
Positive social support from other family	0.00	−0.03, 0.02
Positive social support from friends	0.03	0.00, 0.06
Social strain from spouse	−0.06	−0.11, −0.01*
Social strain from children	−0.07	−0.10, −0.03***
Social strain from other family	−0.02	−0.05, 0.01
Social strain from friends	0.01	−0.03, 0.04
Volunteer		
0 h/year	Reference	Reference
0–49 h/year	0.05	−0.02, 0.12
50–99 h/year	0.02	−0.06, 0.10
100–199 h/year	0.10	−0.01, 0.20
>200 h/year	0.11	0.01, 0.20*
Helping friends/neighbors/relatives		
0 h/year	Reference	Reference
1–49 h/year	0.04	−0.01, 0.09
50–99 h/year	0.03	−0.03, 0.10
100–199 h/year	0.04	−0.06, 0.14
>200 h	0.12	0.03, 0.21**
Religious service attendance		
Not at all	Reference	Reference
<1×/week	0.03	−0.02, 0.09
>1×/week	−0.01	−0.07, 0.06
Social status ladder	0.07	0.03, 0.11**
Change in social status ladder		
Moved down	Reference	Reference
No change	0.08	0.01, 0.16*
Moved up	0.12	0.04, 0.20**
In labor force	0.07	0.00, 0.15*

*Notes*: The analytic sample was restricted to those who had participated in the pre‐baseline wave (2006 or 2008). Multiple imputation was performed to impute missing data on the exposures, covariates, and outcome. Candidate antecedents were assessed, one at a time, in wave 2 (2010/2012), and the outcome (sense of control) was assessed in wave 3 (2014/2016). The following covariates were controlled for at wave 1 (2006/2008): sociodemographic characteristics (age, sex, race/ethnicity, marital status, income, total wealth, level of education, health insurance, geographic region), personality factors (openness, conscientiousness, extraversion, agreeableness, neuroticism), childhood abuse, and all of the predictor variables, including health behaviors (physical activity, smoking, heavy drinking, sleep problems), physical health (heart disease, cancer, stroke, arthritis, hypertension, overweight/obese, diabetes, lung disease, chronic pain, hearing, eyesight, self‐rated health, physical functioning limitations, cognitive impairment), social factors (living with spouse, frequency of contact with children, frequency of contact with other family, frequency of contact with friends, loneliness, closeness with spouse, number of close children, number of close other family, number of close friends, positive social support from spouse, positive social support from children, positive social support from friends, positive social support from other family, social strain from spouse, social strain from children, social strain from other family, social strain from friends, religious service attendance, volunteering, helping friends/neighbors/relatives, perceived social status, change in perceived social status, in labor force), psychological well‐being factors (life satisfaction, positive affect, purpose in life, optimism, health mastery, financial mastery), psychological distress (depressive symptoms, hopelessness, negative affect, anxiety, trait anger, state anger, daily discrimination, major discrimination, cynical hostility, stressful life events, financial strain), and pre‐baseline values of the outcome (sense of control). All continuous candidate antecedents were standardized (mean = 0; standard deviation = 1). An exposure‐wide analytic approach was used, and a separate model for each exposure was run. Higher scores indicate higher sense of control. The *p* value cutoff for Bonferroni correction is *p* = .05/59 predictors = *p* < .00084.

Abbreviations: CI, confidence interval; RR, risk ratio.

* *p* < .05 before Bonferroni correction.

** *p* < .01 before Bonferroni correction.

*** *p* < .05 after Bonferroni correction.

Almost all of the psychological factors (15 out of 18 predictors) were associated with subsequent sense of control. Among psychological well‐being factors, positive affect (*β* = 0.15, 95% CI = 0.11, 0.18) and purpose in life (*β* = 0.14, 95% CI = 0.08, 0.19) were most strongly associated with a higher subsequent sense of control. Among psychological distress factors, hopelessness (*β* = −0.14, 95% CI = −0.18, −0.11) and negative affect (*β* = −0.11, 95% CI = −0.13, −0.09) were most strongly associated with a lower subsequent sense of control. For social factors, 9 out of 22 predictors were associated with subsequent sense of control. Moving up in social status (*β* = 0.12, 95% CI = 0.04, 0.20), and volunteering ≥200 hours/year (*β* = 0.11, 95% CI = 0.01, 0.20) were most strongly associated with higher subsequent sense of control.

### Additional analyses

First, E‐values indicated that many of the observed associations were potentially somewhat robust to unmeasured confounding (Table 3). For example, for purpose in life, an unmeasured confounder that was associated with both sense of control and purpose in life by risk ratios of 1.53 each (above and beyond the covariates already adjusted for) could explain away the association, but a weaker joint confounder associations could not. Further, to shift the CI to include the null, an unmeasured confounder associated with both sense of control and purpose in life by risk ratios of 1.40 each could explain away the association, but weaker joint confounder associations could not. This particular association is thus at least moderately robust to potential unmeasured confounding. However, in other cases, a combination of unmeasured confounding and statistical uncertainty might suffice to explain away the results. Second, we also conducted additional analyses that separately evaluated mastery and perceived constraints as separate outcomes. Results from analyses evaluating mastery as the outcome (see Table A3), and perceived constraints as the outcome (see Table A4) revealed several overlapping predictors that were significant. These overlapping predictors include self‐rated health, positive affect, life satisfaction, optimism, purpose in life, health mastery, financial mastery, hopelessness, negative affect, anxiety, daily discrimination, loneliness, positive social support from children, no change in social status ladder. Additionally, the analyses identified predictors specific to mastery such as sleep problems, physical functioning limitations, eyesight, depressive symptoms, stressful life events, living with a spouse, frequent contact with other family >3x/Week, helping friends/neighbors/relatives >200 h/year, and in the labor force. Conversely, predictors specific to perceived constraints include arthritis, overweight/obese, trait anger, state anger, cynical hostility, social strain from spouse, social strain from children, social strain from other family, volunteering ≥200 h/year. Third, we conducted sensitivity analyses that did not standardize any of the variables (Table A5). When comparing the original results to results from the sensitivity analysis, the general pattern of results remained consistent. However, some effect sizes were slightly attenuated. For example, when unstandardized variables were used, the strength of the associations for many psychological well‐being predictors slightly decreased, while associations with many psychological distress factors slightly increased. However, these changes were small and varied in both direction and magnitude. However, the overall significance and direction of these relationships persisted.

**TABLE 3:** Robustness to unmeasured confounding (E‐values) for the associations between candidate predictors and subsequent sense of control (*N* = 13,771)^a^.

	Effect estimate^b^	Confidence interval limit^c^
Health behaviors		
Frequent physical activity	1.11	1.00
Smoking	1.23	1.00
Heavy drinking	1.11	1.00
Sleep problems	1.27	1.09
Physical health		
Number of physical conditions	1.23	1.00
Diabetes	1.23	1.00
Hypertension	1.00	1.00
Stroke	1.23	1.00
Cancer	1.20	1.00
Heart disease	1.00	1.00
Lung disease	1.16	1.00
Arthritis	1.33	1.00
Overweight/obese	1.33	1.00
Physical functioning limitations	1.39	1.16
Cognitive impairment	1.20	1.00
Chronic pain	1.27	1.00
Self‐rated health	1.36	1.24
Hearing	1.11	1.00
Eyesight	1.23	1.11
Psychological well‐being		
Positive affect	1.56	1.47
Life satisfaction	1.42	1.33
Optimism	1.50	1.42
Purpose in life	1.53	1.40
Health mastery	1.42	1.34
Financial mastery	1.42	1.34
Psychological distress		
Depression	1.42	1.12
Depressive symptoms	1.27	1.14
Hopelessness	1.53	1.45
Negative affect	1.45	1.39
Anxiety	1.42	1.33
Trait anger	1.27	1.18
State anger	1.20	1.10
Cynical hostility	1.30	1.22
Stressful life events	1.16	1.00
Financial strain	1.27	1.14
Daily discrimination	1.30	1.22
Major discrimination	1.11	1.00
Social factors		
Loneliness	1.36	1.28
Living with spouse	1.36	1.02
Contact children		
<Every few months	Reference	Reference
1–2×/month	1.30	1.00
1–2×/week	1.33	1.00
>3×/week	1.39	1.00
Contact other family		
<Every few months	Reference	Reference
1–2×/month	1.20	1.00
1–2×/week	1.16	1.00
>3×/week	1.16	1.00
Contact friends		
<Every few months	Reference	Reference
1–2×/month	1.27	1.00
1–2×/week	1.30	1.00
>3×/week	1.39	1.15
Closeness with spouse	1.11	1.00
Number of close children	1.11	1.00
Number of close other family	1.11	1.00
Number of close friends	1.16	1.00
Positive social support from spouse	1.23	1.00
Positive social support from children	1.30	1.19
Positive social support from other family	1.00	1.00
Positive social support from friends	1.11	1.00
Social strain from spouse	1.30	1.15
Social strain from children	1.33	1.23
Social strain from other family	1.16	1.00
Social strain from friends	1.10	1.00
Volunteer		
0 h/year	Reference	Reference
0–49 h/year	1.27	1.00
50–99 h/year	1.16	1.00
100–199 h/year	1.42	1.07
>200 h/year	1.45	1.14
Helping friends/neighbors/relatives		
0 h/year	Reference	Reference
1–49 h/year	1.23	1.00
50–99 h/year	1.20	1.00
100–199 h/year	1.27	1.00
>200 h	1.47	1.29
Religious service attendance		
Not at All	Reference	Reference
<1×/week	1.20	1.00
>1×/week	1.11	1.00
Social status ladder	1.33	1.21
Change in social status ladder		
Moved down	Reference	Reference
No change	1.36	1.09
Moved up	1.47	1.23
In labor force	1.33	1.05

^a^ See VanderWeele and Ding (2017) for the formula for calculating E‐values.

^b^ The E‐values for effect estimates are the minimum strength of association on the risk ratio scale that an unmeasured confounder would need to have with both the exposure and the outcome to fully explain away the observed association between the exposure and outcome, conditional on the measured covariates.

^c^ The E‐values for the limit of the 95% confidence interval (CI) closest to the null denote the minimum strength of association on the risk ratio scale that an unmeasured confounder would need to have with both the exposure and the outcome to shift the confidence interval to include the null value, conditional on the measured covariates.

## DISCUSSION

In a large, prospective, diverse, and national representative sample of U.S. adults aged >50, we examined how *changes in* 59 candidate predictors (e.g., physical health, health behavior, and psychosocial factors) were associated with subsequent sense of control four years later. Our findings converge with previous studies that observed how psychological factors (e.g., increased: life satisfaction, decreased depressive symptoms) (Infurna et al., 2011; Infurna & Okun, 2015), social factors (e.g., increased: volunteering, perceived emotional support from one's spouse and family; decreased: loneliness) (Drewelies et al., 2017; Gerstorf et al., 2011; Infurna & Okun, 2015), health factors (increased: self‐rated health, decreased: functional limitations) (Drewelies et al., 2017; Infurna et al., 2011; Infurna & Okun, 2015), were positively associated with sense of control. However, our results also diverge with results from prior studies which observed other factors that were associated with a decreased sense of control (e.g., composite health variables created by adding together several specific health conditions, religious service attendance, increased social support) (Drewelies et al., 2017; Gerstorf et al., 2011; Infurna et al., 2011; Penninx et al., 2000).

Several potential reasons might explain our diverging results, including differences in: 1) composition of the study sample (e.g., older vs. younger adult samples, healthy sample vs. sample with a specific health condition), 2) study design (e.g., varying follow‐up periods, simultaneous controlling/covarying of predictors), 3) measurement/categorization of exposures (e.g., stable levels of predictors vs. *changes* in predictors) and the sense of control outcome (e.g., use of different scales: Pearlin Mastery Scale, relevant items from other scales (e.g., Dispositional Hope Scale)), 4) number of covariates (e.g., differences in specific questionnaires/items, including fewer vs. a larger range of covariates) and measurement of covariates.

Some of our findings, might be understood through a broader application of Bandura's theory (Bandura, 1997), which posits that a person's sense of control, involves beliefs about personal mastery (i.e., self‐efficacy) and perceived constraints (i.e., outcome expectations) and is influenced by 1) past experiences with one's performance and abilities, 2) feedback from interactions with others, and 3) affective and emotional reactions. Through the lens of experiences with one's performance and abilities, we observed that several candidate health factors (e.g., lower: physical functioning, eyesight) were associated with a decreased sense of control. These factors degrade people's ability to successfully navigate their environments (e.g., transportation, meal preparation) and pursue meaningful goals; compounded over time, these challenging experiences might accumulate and diminish a person's sense of control. Interestingly, other specific health conditions (e.g., stroke, arthritis) and health behaviors (e.g., heavy drinking) were not associated with subsequent sense of mastery which is an area needing further research. When considering the importance of feedback from interactions with others, we observed that several candidate social factors were associated with a higher sense of control (e.g., contacting friends ≥3x/week, positive social support from children) and lower sense of control (e.g., loneliness, social strain from spouse, social strain from children). Further, social factors have been theorized as critical sources of sense of control because they provide several critical resources (e.g., instrumental‐, emotional‐, informational‐support) that enhance people's capacity to exercise control over obtaining desired outcomes (Antonucci, 2001; Bandura, 1997). Finally, when considering affective and emotional reactions, we observed that several psychological well‐being candidates (e.g., purpose in life, optimism) and psychological distress candidates (e.g., depressive symptoms, hopelessness, negative affect) were associated with higher and lower sense of control, respectively. These psychological factors play critical roles in enhancing, and eroding, people's motivations and behaviors when pursuing goals, which in turn might influence a person's overarching sense of control. While our study did not directly probe the distinct components of Bandura's framework, it echoes the central premise that an individual's sense of control is intricately linked with their experiential, social, and psychological contexts.

We identified some predictors with relatively larger effect sizes. Among the psychological well‐being predictors, positive affect (β = 0.15), purpose in life (β = 0.14), and optimism (β = 0.13), emerged as the strongest positive predictors​​ of sense of control. These were followed by: life satisfaction (β = 0.10), financial mastery (β = 0.10), and health mastery (β = 0.10). Among social factors, helping friends/neighbors/relatives >200 hours/year (β = 0.12) and volunteering >200 hours/year (β = 0.11) emerged as relatively strong positive predictors. There were also other social factors that appeared to have smaller, but still notable influences on sense of control (e.g., a perception of moving up on the social status ladder (β = 0.12), loneliness (β = −0.08), and contact with friends >3x/Week (β = 0.09)). Conversely, among psychological distress factors, hopelessness (β = −0.14) was the most potent negative predictor, followed by negative affect (β = −0.11) and anxiety (β = −0.10). Surprisingly most physical health conditions and health behaviors did not seem to influence sense of control. These findings indicate that fostering all facets of psychological well‐being and higher amounts of prosocial efforts (i.e., 2 or more hours per week of informally helping friends/neighbors/relatives and/or formal volunteering), as well as decreasing certain facets of psychological distress might be equally powerful and complementary pathways to enhancing a person's sense of control. Further, when comparing results from additional analyses that separately evaluated mastery vs. perceived constraints as the outcomes, we observed several overlapping predictors that were significant in opposite directions, as expected. However, we also observed many unique predictors. Overall, mastery appears to be more influenced by factors that enhance an individual's internal sense of control and self‐efficacy, such as psychological well‐being factors, good health, and supportive relationships. These elements foster a belief in personal agency and competence. In contrast, perceived constraint appears to be more influenced by factors that induce stress and limit personal freedom, such as chronic health conditions, various forms of anger and hostility, and negative social interactions. These factors reinforce a sense of powerlessness and should be further studied and carefully considered in future intervention efforts.

Our study had several limitations. First, many physical health outcomes and health behaviors were self‐reported and thus are vulnerable to self‐report bias. Study participants, however, were not aware of the study's hypothesis at the time of data collection. Moreover, this concern is also partly mitigated by our controlling for these same variables in the pre‐baseline wave. Second, there is potential for unmeasured confounding. Yet, we were able to evaluate this concern specifically with robust covariate adjustment, use of a prospective design, and E‐value analyses. Third, temporal factors should be taken into consideration when interpreting findings. Some factors that were associated with subsequent sense of control in our study (e.g., depressive symptoms) are subject to acute intra‐individual changes, and their impact on sense of control could be transitory. Thus, a 4‐year interval between measurements may be too long to capture such a phenomenon. Fourth, many of the effect sizes of any given predictor were relatively modest, often corresponding to only a 0.05 or a 0.15 difference in subsequent sense of control. Notably, there was a wide range of predictors associated with modest changes in sense of control, which suggests a multi‐faceted approach may be most effective when attempting to enhance a person's sense of control. Additionally, small effects can have important consequences when accumulated over time and considered at the population‐level (Götz et al., 2022). Fifth, longitudinal studies, like HRS, suffer from attrition which could impact the results. However, we used multiple imputation because it provides a potentially more accurate approach than other methods of handling missing data (Groenwold et al., 2012; Moons et al., 2006; Sterne et al., 2009). Additionally, we also compared those who were lost to follow‐up vs. those who stayed in HRS. Compared to those who were lost to follow‐up, people who stayed in HRS were generally healthier, wealthier, more highly educated, and had better health behaviors and mental health. However, we controlled for all of these characteristics, and a robust range of other covariates, in all of our analyses. This helps mitigate bias introduced if these characteristics are associated with both attrition and the outcomes. Sixth, we conducted this exploratory study due to the limited research that has examined a wide array of potential predictors influencing sense of control. Our aim was to broadly map the landscape of factors associated with changes in sense of control over time. This broad aim allowed us to examine several predictors in an exploratory manner, and to identify many new candidate predictors. However, this strength of our study (our ability to evaluate a large range of predictors) was also a weakness (our capacity to delve deeply into any specific predictor was limited). For example, our predictors varied across multiple dimensions, including modifiability, proximity to sense of control, categorical vs. continuous nature, linear vs. potential non‐linear associations, and the dual nature of some factors as both resources and liabilities (e.g., social support, typically a resource, may become a liability if a person becomes excessively dependent on others). Additionally, overlaps among some predictors (e.g., depression and negative affect) and the inclusion of both domain‐specific and general predictors (e.g., health mastery vs. mastery) underscores the complexity of our analysis. These nuances highlight the need for future research that will explore these aspects more thoroughly. Ultimately, this study builds upon the seminal work of others and marks another step toward identifying pathways to enhance the sense of control. The current study also had several strengths, such as the use of a prospective, large, diverse, and national representative sample of U.S. adults aged >50. Further, we evaluated several novel predictors that have been understudied, or not previously studied. We also evaluated all predictors within the same study, allowing us to compare effect sizes.

Policies aimed at enhancing sense of control (on both the individual‐ and structural‐levels) may be a promising and innovative way to improve a wide range of health and well‐being outcomes for our rapidly growing older adult population. However, a key challenge in advancing intervention development is the identification of antecedents that predict sense of control. While many potential targets have been identified, determining which will have the most significant influence remains complex. Our results highlight potential building blocks that can be targeted as we continue developing, refining, and potentially deploying interventions that aim to enhance sense of control. Prioritizing these targets effectively will be crucial to maximizing the impact of future interventions.

## SOURCES OF FUNDING

This work was supported by grants from the Michael Smith Health Research BC and the John Templeton Foundation. The funding source had no impact in the study design; in the collection, analysis, and interpretation of data; in the writing of the report; or in the decision to submit the article for publication.

## AUTHOR CONTRIBUTIONS

E.S.K. had full access to all the data in the study and took responsibility for the integrity of the data and the accuracy of the data analysis; all authors contributed to the study concept and design; all authors contributed to the acquisition, analysis, or interpretation of data; all authors contributed to drafting the manuscript; all authors contributed to critical revision of the manuscript for important intellectual content.

## CONFLICT OF INTEREST STATEMENT

Eric S. Kim has worked as a consultant with AARP and UnitedHealth Group. Tyler J. VanderWeele reports receiving licensing fees from Flerish Inc. and Flourishing Metrics.

## ETHICS STATEMENT

The HRS has been approved by several ethics committees, including the University of Michigan IRB. Further, informed consent was obtained from all HRS respondents.

## DATA SHARING

All data are available here: https://hrs.isr.umich.edu/data-products and the authors will share syntax upon request.

## Data Availability

Data and code are available upon reasonable request.

## APPENDIX A TEXT 1 ASSESSMENT OF PREDICTORS

Reference Group.

The reference group was the healthiest group for all binary outcomes unless otherwise noted.

Health Behaviors.

*Frequent physical activity*. Based on prior research, a binary physical activity variable was created: ≥1x/week of vigorous or moderate exercise was considered frequent physical activity, while <1x/week of vigorous or moderate exercise was the reference group.^1^ Participants indicated the frequency (i.e., response categories: daily, >1x/week, 1x/week, 1‐3x/month, hardly ever or never) with which they engaged in vigorous (e.g., running, swimming, aerobics), moderate (e.g., gardening, dancing, walking at a moderate pace), and light (e.g., vacuuming, laundry) activities over the past 12 months.

*Smoking*. Participants were asked (yes/no), “Do you smoke cigarettes now?” to assess current smoking status. The reference group was “no” smoking.

*Binge drinking*. Following the National Institute on Alcohol Abuse and Alcoholism guidelines,^2^ binge drinking was defined as >14 for drinks/week for men and >7 drinks/week for women. Alcohol consumption was measured by multiplying the number of days/week that alcohol was consumed x number of drinks/day, which resulted in the number of drinks/week. Participants not in this alcohol consumption range were classified as non‐binge drinkers (the reference group).

*Sleep problems*. Participants completed the 4‐item Jenkins Sleep Questionnaire, a widely used and validated screening instrument for assessing sleep complaints and insomnia symptoms.^3^ Response categories included “most of the time,” “sometimes,” and “rarely or never.” Having sleep problems was defined as reporting: “most of the time” for any of the three negatively worded items (e.g., “How often do you have trouble falling asleep?”) and “rarely or never” to the one positively worded item (i.e., “feel really rested when you wake up in the morning”). Participants were considered unhealthy (i.e., having sleep problems) if they reported one or more sleep problems. The sleep questionnaire was only administered every other wave. Thus, sleep data was imputed for half of the sample. Imputed and complete‐case analyses showed similar estimates.

Physical Health.

*Physical health conditions*. Participants self‐reported (yes/no) if they were ever told by a healthcare provider that they had the following conditions: 1) diabetes, 2) hypertension, 3) stroke, 4) cancer, 5) heart disease, 6) lung disease, or 7) arthritis. The HRS has demonstrated validity and reliability of self‐reported chronic conditions.^4^

*Overweight/obese*. Body mass index (BMI) was derived from self‐reported height and weight. It was calculated as weight/height^2^ (kg/m^2^). A BMI of ≥25 kg/m^2^ was considered as overweight/obese.^5^

*Number of chronic conditions*. To create a score for the number of chronic conditions, a summary score was calculated by summing the number of reported conditions. This measure included the 7 chronic conditions above and overweight/obesity (range 0–8).

*Physical functioning limitations*. Physical functioning limitations were assessed using items from scales developed by Rosow and Breslau (1966), Nagi (1976), Katz, Ford, Moskowitz, Jackson, and Jaffe (1963), and Lawton and Brody (1969).^6–9^ A total of 15 questions about physical functioning (e.g., walking several blocks, climbing one flight of stairs, pushing or pulling large objects, lifting or carrying 10 pounds, getting up from a chair, reaching or extending arms up, stooping, kneeling, or crouching, sitting for 2 hours) and activities of daily living (e.g., walking across a room, dressing, eating, bathing, getting in/out bed, using the toilet, picking up a dime) were included. Participants were classified as having “physical functioning limitations” if they reported >4 limitations with physical functioning, while participants who reported ≤4 limitations were considered “normal” (the reference group). This criterion was determined by identifying the physical function score where 75% of participants could be considered as having healthy physical function at baseline in the HRS sample.

*Cognitive impairment*. The HRS cognitive functioning assessment^10,11^ was adapted from the modified Telephone Interview for Cognitive Status (TICS‐M). The assessment included an immediate and delayed 10‐noun free recall test, a serial 7 subtraction test, and a backward count 20 test (27‐point scale overall). This assessment tool has been shown to have high sensitivity and specificity when assessing cognitive impairment in older adults. The cut‐off points used in this study were derived from previous research on cognitive impairment in HRS.^12,13^ Participants who scored 0–11 (on the 27‐point scale) were classified as having “cognitive impairment,” while participants who scored ≥12 were classified as “normal” (the reference group). HRS reports contain further information about these cognitive assessments.^10,11^

*Chronic pain*. Participants were asked (yes/no), “Are you often troubled with pain?” The reference group was “no” pain.

*Self‐rated health*. Participants were asked, “Would you say your health is excellent, very good, good, fair, or poor?” on a 5‐point scale (reverse coded with higher scores indicating higher self‐rated health).

*Hearing*. Participants were asked, “Is your hearing excellent, very good, good, fair, or poor (using a hearing aid as usual)?” on a 5‐point Likert scale (reverse coded with higher values indicating better hearing). Self‐report measures of hearing have been found to be reliable measures of hearing impairment.^14^

*Eyesight*. Participants were asked, “Is your eyesight excellent, very good, good, fair, or poor using glasses or corrective lenses as usual?” Response categories were as follows: 1) Excellent, 2) Very good, 3) Good, 4) Fair, 5) Poor, or 6) Legally blind. Responses were reverse‐coded such that higher values were associated with better eyesight.

Psychological Well‐Being.

*Positive affect*. Positive affect was measured (in 2006 only) with a 6‐item scale^15–17^ originally developed for use in the Midlife in the United States Study. The scale assessed how often the participant felt “cheerful,” “in good spirits,” “extremely happy,” “calm and peaceful,” “satisfied,” and “full of life” over the past 30 days. Response categories ranged from 1 (all of the time) to 5 (none of the time). Responses were reverse scored, so that a higher score indicated higher positive affect. An overall score was derived by averaging responses across all 6 items (α = 0.91 in 2006, range = 1 to 5). After the 2006 wave, the HRS switched to a more expansive measure of positive affect based on the Positive and Negative Affect Schedule (PANAS‐X).^18^ It included the following 13 items: determined, enthusiastic, active, proud, interested, happy, attentive, content, inspired, hopeful, alert, calm, and excited. An overall score was derived by averaging responses across all 13 items (α = 0.92 in 2008, range = 1 to 5). A limitation of this study is that affect was measured in a different way during only the first wave of the study. However, scores were standardized and both the prior and current measures of affect operate very similarly (e.g., similar correlations with other variables, similar pattern of descriptive statistics).

*Life satisfaction*. Life satisfaction was assessed with the 5‐item Satisfaction with Life Scale.^19^ The scale has shown excellent psychometric properties in prior work. Using a 7‐point Likert scale (from 1 (strongly disagree) to 7 (strongly agree)), participants were asked the extent to which they agreed with statements such as, “In most ways my life is close to ideal.” Responses to all items were averaged to create a composite score, with higher scores indicating higher life satisfaction (α = 0.88, range 1–7).

*Optimism*. Optimism was assessed with the Life Orientation Test‐Revised (LOT‐R), which has good discriminant and convergent validity, as well as good reliability.^20^ Using a 6‐point Likert scale (from 1 (strongly disagree) to 6 (strongly agree)), participants were asked the degree to which they agreed with statements such as, “In uncertain times, I usually expect the best.” Negatively worded items were reverse coded and responses to all items were averaged to create an overall score, with higher scores indicating higher optimism (α = 0.75, range 1–6).

*Purpose in life*. Purpose in life was assessed with a 7‐item purpose in life subscale from Ryff's Psychological Well‐Being Scale.^21^ The 7‐item subscale has been validated in prior work and has shown good psychometric properties.^22^ Using a 6‐point Likert scale (from 1 (strongly disagree) to 6 (strongly agree)), participants were asked the degree to which they agreed with statements such as, “I have a sense of direction and purpose in my life.” Negatively worded items were reverse coded and all items were averaged to create a composite score, with higher scores indicating higher purpose (α = 0.77, range 1–6).

*Mastery*. Mastery was assessed with 5‐items derived from Lachman and Weaver (1998). The measure has good discriminant and convergent validity, and good reliability.^23^ Using a 6‐point Likert scale (from 1 (strongly disagree) to 6 (strongly agree)), participants were asked the degree to which they agreed with statements such as, “I can do just about anything I really set my mind to.” All items were averaged to create a composite score, with higher scores indicating higher mastery (α = 0.90, range 1–6).

*Health mastery*. Participants were asked, “How would you rate the amount of control you have over your health these days?” on a 0 (“no control at all”) to 10 (“very much control”) scale.

*Financial mastery*. Participants were asked, “How would you rate the amount of control you have over your financial situation these days?” on a 0 (“no control at all”) to 10 (“very much control”) scale.

Psychological Distress.

*Depressive symptoms and depression*. Depressive symptoms were measured using The Center for Epidemiologic Studies Depression Scale (CESD).^24^ This scale has been validated in the HRS.^25^ Participants indicated the presence of 8 depressive symptoms (e.g., “Much of the time during the past week, I felt depressed”) over the past week (yes/no). All items were summed, with higher scores indicating higher depressive symptoms (α = 0.80, range 0–8). Participants with scores of ≥4 were classified as having depression, as done previously (no depression was the reference group).^25^ Prior work has suggested that the cutoff value of 4 would produce results similar to the 16‐item cutoff when using the full (20‐item) CESD scale.^25^

*Hopelessness*. Hopelessness was assessed with a 4‐item questionnaire from two previously validated scales.^26,27^ Using a 6‐point Likert scale (from 1 (strongly disagree) to 6 (strongly agree)), participants were asked the degree to which they agree with statements such as, “The future seems hopeless to me and I can't believe that things are changing for the better.” All items were averaged to create a composite score (α = 0.86, range 1–6), with higher scores indicating more hopelessness.

*Negative affect*. Negative affect was measured (in 2006 only) with a 6‐item scale originally developed for use in the Midlife in the United States Study.^15–17^ The scale assessed how often the participant felt “so depressed that nothing could cheer you up,” “hopeless,” “restless or fidgety,” “that everything was an effort,” “worthless,” and “nervous” over the past 30 days. Response categories ranged from 1 (all of the time) to 5 (none of the time). Responses were reverse scored, so that a higher score indicated higher negative affect. An overall score was derived by averaging responses across all 6 items (α = 0.87, range = 1 to 5). After the 2006 wave, the HRS switched to a more expansive measure of negative affect based on the Positive and Negative Affect Schedule (PANAS‐X).^18^ It included the following 12 items: afraid, upset, guilty, scared, frustrated, bored, hostile, jittery, ashamed, nervous, sad, and distressed. An overall score was derived by averaging responses across all 12 items (α = 0.89, range = 1 to 5). A limitation of this study is that affect was measured in a different way during only the first wave of the study. However, scores were standardized and both the prior and current measures of affect operate very similarly (e.g., similar correlations with other variables, similar pattern of descriptive statistics).

*Perceived constraints*. Perceived constraints were assessed with 5 other items derived from Lachman and Weaver (1998), and this measure has good discriminant and convergent validity, as well as good reliability.^23^ Using a 6‐point Likert scale (from 1 (strongly disagree) to 6 (strongly agree)), participants were asked the degree to which they agreed with statements such as, “What happens in my life is often beyond my control.” All items were averaged to create an overall score, with higher scores indicating a higher sense of constraints on personal control (α = 0.87, range 1–6).

*Anxiety*. Anxiety was assessed using 5 of the 21 items in the Beck Anxiety Inventory (BAI).^28^ This inventory has been shown to differentiate between symptoms of depression and anxiety and has been validated in older adults.^29^ Participants were asked, “How often did you feel that way during the past week.” 1) “I had fear of the worst happening,” 2) “I was nervous,” 3) “I felt my hands trembling,” 4) “I had a fear of dying,” and 5) “I felt faint,” and could respond with 1 of 4 categories: 1) Never, 2) Hardly ever, 3) Some of the time, 4) Most of the time. The five responses were averaged, with higher scores indicating greater anxiety symptoms (α = 0.81, range 1–4). The final score was set to missing if more than 2 of the individual items were missing.

*Trait anger and state anger*. Trait anger (anger‐in) and state anger (anger‐out) are the two dimensions along which the Spielberger Anger Expression Scale (STAX) measures anger.^30^ These two dimensions have been shown to be separate factors that are modestly correlated through a principal factor analysis with Promax rotation.^31^ Trait anger is the predisposition to respond with anger across a variety of situations. To measure this variable, participants were asked to respond to four statements such as, “When I am feeling angry or mad, I keep things in.” State anger is a temporary behavioral reaction of anger and was measured through seven statements including, “When I am feeling angry or mad, I strike out at whatever infuriates me.” Participants gave responses on a 4‐point Likert scale for each item: 1) Almost never, 2) Sometimes, 3) Often, and 4) Almost always. Responses were averaged for trait anger (α = 0.80) and state anger (α = 0.82) separately, with higher scores indicating higher trait anger and state anger (range 1–4). If more than 2 values were missing for trait anger or more than 3 values were missing for state anger, the final score was set to missing.

*Cynical hostility*. Cynical hostility was measured using 5 items from the Cook‐Medley Hostility Inventory.^32^ The items were as follows: 1) “Most people dislike putting themselves out to help other people,” 2) “Most people will use somewhat unfair means to gain profit or an advantage rather than lose it,” 3) “No one cares much what happens to you,” 4) “I think most people would lie in order to get ahead,” and 5) “I commonly wonder what hidden reasons another person may have for doing something nice for me. The first statement was written as, “Most people inwardly dislike putting themselves out to help other people” in the 2006 and 2008 questionnaire before being changed from 2010 onwards. Participants responded on a 6‐point Likert scale (from 1 (strongly disagree) to 6 (strongly agree)). The scores were averaged (α = 0.78, range 1–6), with higher scores indicating higher cynical hostility. The index was set to missing if more than three items were missing.

*Stressful life events*. Stressful life events were measured using 5 questions that have been used in other widely‐used self‐report measures of life stress.^33^ Items included questions such as, “Have you been unemployed and looking for work for longer than 3 months at some point in the past five years?” While the questionnaire in 2008 onwards asked an additional question of, “Have you been the victim of fraud in the past five years?”, this was not included for the purposes of the present study to maintain consistency (since it was not included in the 2006 questionnaire). Participants answered each question with a yes or no. Responses (0 = no, 1 = yes) were summed, with higher values indicating a higher number of stressful life events.

*Financial strain*. Respondents were asked, “How difficult is it for (you/your family) to meet monthly payments on (your/your family's) bills?” and response options included: 1) Not at all difficult, 2) Not very difficult, 3) Somewhat difficult, 4) Very difficult, or 5) Completely difficult. Higher scores indicated more financial strain.

*Daily discrimination and major discrimination*. Items measuring daily discrimination and major discrimination were based on prior widely used discrimination assessments.^34–36^ Daily discrimination was measured using 5 items that capture the frequency of the following experiences in the day‐to‐day lives of participants: 1) being treated with less courtesy or respect, 2) receiving poorer service in restaurants or stores, 3) people acting as if you are not smart, 4) people acting as if they are afraid of you, and 5) being threatened or harassed. Participants answered with one of the following response categories: 1) Almost every day, 2) At least once a week, 3) A few times a month, 4) A few times a year, 5) Less than once a year, and 6) Never. Items were reverse‐coded and averaged (α = 0.80, range 1–6) such that higher scores indicated higher daily discrimination. The final score was set to missing if more than 3 items were missing. The item, “You receive poorer service or treatment than other people from doctors or hospitals” (introduced in 2008) was excluded in the present study to maintain consistency as it was not present in the 2006 questionnaire. Major discrimination was measured using 6 items (yes/no) to capture major instances of lifetime discrimination: 1) being unfairly dismissed from a job, 2) not being hired for a job, 3) being unfairly denied a promotion, 4) being prevented from moving to a neighborhood because the realtor refused to sell/rent to you, 5) being unfairly denied a bank loan, and 6) being unfairly stopped by the police. Responses were summed with higher scores indicating more experiences of major discrimination. One item (“Have you ever been unfairly denied health care or treatment?” (introduced in 2008)) was excluded in the present study to maintain consistency as it was not included in the 2006 questionnaire.

Social Factors.

*Loneliness*. Loneliness was assessed with three items from the previously validated UCLA Loneliness Scale.^37^ Participants were asked, “How much of the time do you feel”: 1) “you lack companionship”, 2) “left out”, and 3) “isolated from others”, with response categories ranging from 1 (often) to 3 (hardly ever or never). Responses were reverse scored and averaged, with higher scores indicating higher loneliness (α = 0.80, range 1–3).

*Living with a partner/spouse*. Participants were asked, “Do you have a husband, wife, or partner with whom you live?,” and answered yes/no.

*Frequency of contact with children/other family/friends*. The frequency of contact respondents had with members in their social network was evaluated through 3 items each for contacts who had 1) children, 2) other family, and 3) friends. Participants were asked, “On average, how often do you do each of the following?” 1) “Meet up (include both arranged and chance meetings),” 2) “Speak on the phone,” and 3) “Write or email.” Possible response categories were as follows: 1) Three or more times a week, 2) Once or twice a week, 3) Once or twice a month, 4) Every few months, 5) Once or twice a year, or 6) Less than once a year or never. The responses were re‐coded into the following categories: 0 = Every few months ‐ never, 1 = 1‐2x/month, 2 = 1‐2x/week, and 3 = 3 or more times/week.

*Closeness with spouse*. One's closeness with their spouse, if they had one, was assessed using a single question, “How close is your relationship with your spouse or partner?” Response options included: 1) Very close, 2) Quite close, 3) Not very close, and 4) Not at all close. Responses were reverse‐coded to range from 1 (not at all close) to 4 (very close).

*Number of close children, close other family, close friends*. The quantity of close social ties was measured through the following 3 items: 1) “How many of your children would you say you have a close relationship with?”, 2) “How many of these family members would you say you have a close relationship with?”, and 3) “How many of your friends would you say you have a close relationship with?”

*Positive social support from spouse, children, other family, friends + Social strain from spouse, children, other family, friends*. The positive social support and negative social strain associated with close relationships were assessed using three and four items, respectively, for each category of social ties. These items were based on those used in previous studies on social support.^38–40^ Items assessing positive social support were as follows: 1) “How much do they really understand the way you feel about things?”, 2) “How much can you rely on them if you have a serious problem?” and 3) “How much can you open up to them if you need to talk about your worries?” The 4 items assessing social strain were: 1) “How often do they make too many demands on you?”, 2) “How much do they criticize you?”, 3) “How much do they let you down when you are counting on them?” and 4) “How much do they get on your nerves?” Response options for all 7 questions included: 1) A lot, 2) Some, 3) A little, or 4) Not at all. Scores were reverse‐coded and then averaged to create separate indexes for positive social support and negative social strain. Higher values indicated more positive social support or more social strain. If more than 1 or more than 2 items were missing, the value was set to missing for positive social support and social strain, respectively. This was done for positive social support from spouse (α = 0.81), children (α = 0.82), other family (α = 0.82), and friends (α = 0.84), as well as negative social strain from spouse (α = 0.78), children (α = 0.77), other family (α = 0.78), and friends (α = 0.75).

*Volunteering*. Respondents were asked, “Have you spent any time in the past 12 months doing volunteer work for religious, educational, health‐related or other charitable organizations?” If they answered yes to this question, respondents were asked how hours they volunteered. Responses were coded as 0 = 0 hours, 1 = 1–49 hours, 2 = 50–99 hours, 3 = 100–199 hours, and 4 ≥ 200 hours. Higher values indicated a greater amount of time spent volunteering.

*Helping friends/neighbors/relatives*. Respondents were asked, “Have you spent any time in the past 12 months helping friends, neighbors, or relatives who did not live with you and did not pay you for the help?” If they answered yes to this question, respondents were asked how many hours they volunteered. Responses were coded as 0 = 0 hours, 1 = 0–49 hours, 2 = 50–99 hours, 3 = 100–199 hours, and 4 = ≥ 200 hours. Higher values indicated a greater amount of time spent helping others.

*Religious service attendance*. Participants were asked, “About how often have you attended religious services during the past year?” Possible response categories were as follows: 1) more than once a week, 2) once a week, 3) two or three times a month, 4) one or more times a year, or 5) not at all. Response categories of 1 or 2 were redefined as “>or = 1x/week.” Response categories of 3 or 4 were redefined as “<1x/week.” A response category of 5 was consistently defined as “Not at all.”

*Social status ladder and change in social status ladder*. The MacArthur scale of subjective social status was used to evaluate an individual's own position on the social ladder.^41^ Participants were asked to think of a ladder on which the people at the top were best off and those at the bottom were worst off based on money, education level, and job quality (e.g., having one of the best jobs vs. having the worst jobs or no job). The first item asked respondents to place themselves on the ladder (range: 1–10). The second item asked, “Has your position on the ladder changed within the last two years?” Participants could answer 1) Yes, I have moved up, 2) Yes, I have moved down or 3) No, my position has not changed. Responses were re‐coded into the following categories: 1 = downward movement, 2 = no change, and 3 = upward movement.

*In labor force*. Participants were asked, “Are you currently working?” An answer of 1 indicated “yes” while a 5 indicated “no”. Responses were recoded such that 1 = “In labor force” and 0 = “Not in labor force”.

## APPENDIX B TEXT 2. PROOF ILLUSTRATING HOW ADJUSTING FOR PRE‐BASELINE LEVELS OF EXPOSURE CAN HELP US EVALUATE HOW “CHANGES” IN EXPOSURE ARE ASSOCIATED WITH SUBSEQUENT SENSE OF CONTROL OVER TIME

Let Y be the sense of control outcome in 2014/2016, A_1_ the exposure under consideration in 2010/2012, A_0_ the prior level of exposure in 2006/2008, and C the set of all other covariates in 2006/2008.

For a continuous outcome, the regression model is E[Y|a_0_, a_1_, c] = v + b_0_a_0_ + b_1_a_1_ + b_2_’c. Let Y_a_ denote the potential outcome Y for an individual who has a change in exposure to set A_1_ to a. For an individual with baseline exposure A_0_ = a_0_ and covariates c in 2006/2008, under the no‐confounding (and positivity and consistency) and modeling assumptions, a change in exposure of d points A_0_ = a_0_ to A_1_ = a_0_ + d in 2010/2012, rather than maintaining exposure of A_1_ = a_0_ in 2010/2012, will give rise to an effect (a difference in potential outcomes for Y) of:

$E\left[Y_{a0+d}|\;A_{0}=a_{0},c\right]-E\left[Y_{\text{a0}}|A_{0}=a_{0},c\right]$

$=E\left[Y_{a0+d}|A_{1}=a_{0}+d,A_{0}=a_{0},c\right]-E\left[Y_{\text{a0}}|A_{1}=a_{0},A_{0}=a_{0},c\right]$

$=E\left[Y|A_{1}=a_{0}+d,A_{0}=a_{0},c\right]-E\left[Y|A_{1}=a_{0},A_{0}=a_{0},c\right]$

$=\left[v+b_{0}a_{0}+b_{1}\left(a_{0}+d\right)+{b_{2}}^{’}c\right]-\left[v+b_{0}a_{0}+b_{1}a_{0}+{b_{2}}^{’}c\right]$

$=b_{1}d$

where the first equality is followed by the no‐confounding assumption, the second by consistency, and the third by the statistical model.

Our focus was on how changes in predictors are associated with subsequent changes in sense of control over time. To achieve this, we adjusted for pre‐baseline levels of each predictor to assess the impact of changes in these predictors from pre‐baseline to the baseline wave. The model includes baseline (t1;2010/2012) predictor levels and control for pre‐baseline (t0;2006/2008) predictor levels and covariates. The formula provided illustrates how a change in predictor (from A0 to A1) is associated with a change in sense of control, accounting for pre‐baseline covariates. Specifically, the effect of interest is modeled as the difference in potential outcomes for the sense of control, given a change in predictor of d points, with adjustments for prior predictor levels and covariates. To clarify, our analysis does not use residualized change scores but rather focuses on the association between baseline predictors and subsequent sense of control, adjusting for pre‐baseline predictor levels. This approach allows us to interpret the effect estimates as the association between a change in predictor from the pre‐baseline to the baseline wave and sense of control at the outcome wave, conditional on the predictor and covariates in the pre‐baseline wave. The scores are standardized by subtracting the mean of each variable within timepoint and dividing by the standard deviation of the variable at that time point to aid interpretability. Additionally, the causal effect definition is irrespective of scale, and the estimate for the exposure would be the same even if the pre‐exposure level had been standardized differently or left unstandardized. The interpretation is a change in one standard deviation in the exposure based on the standard deviation at the exposure (not the pre‐exposure) time.

### Appendix Text References

1. Fisher GG, Faul JD, Weir DR, Wallace RB. Documentation of chronic disease measures in the Health and Retirement Study (HRS/AHEAD). Health and Retirement Study. Updated February 10, 2005. Accessed September 19, 2023. https://hrs.isr.umich.edu/sites/default/files/biblio/dr-009.pdf

2. World Health Organization. Physical status: the use and interpretation of anthropometry: report of a WHO expert committee. Published 1995. September 19, 2023. https://apps.who.int/iris/bitstream/handle/10665/37003/WHO_TRS_854.pdf.

3. Rosow I, Breslau N. A Guttman health scale for the aged. *J Gerontol*. 1966;21(4):556–559. doi: 10.1093/geronj/21.4.556

4. Nagi SZ. An epidemiology of disability among adults in the United States. *Milbank Mem Fund Q Health Soc*. 1976;54(4):439–467. doi: 10.2307/3349677

5. Katz S, Ford AB, Moskowitz RW, Jackson BA, Jaffe MW. Studies of illness in the aged: the index of ADL: A standardized measure of biological and psychosocial function. *JAMA*. 1963;185(12):914–919. doi: 10.1001/jama.1963.03060120024016

6. Lawton MP, Brody EM. Assessment of older people: self‐maintaining and instrumental activities of daily living. *Gerontologist*. 1969;9(3):179–186. doi: 10.1093/geront/9.3_Part_1.179

7. Fisher GG, Halimah H, Faul JD, Rodgers WL, Weir DR. Health and Retirement Study imputation of cognitive functioning measures: 1992–2014. Ann Arbor, MI: University of Michigan. Published January 13, 2017. September 19, 2023. https://hrs.isr.umich.edu/sites/default/files/biblio/COGIMPdd.pdf

8. Ofstedal MB, Fisher GG, Herzog AR. Documentation of cognitive functioning measures in the Health and Retirement Study. Ann Arbor, MI: University of Michigan. Published March, 2005. September 19, 2023. https://hrs.isr.umich.edu/sites/default/files/biblio/dr-006.pdf

9. Crimmins EM, Kim JK, Langa KM, Weir DR. Assessment of cognition using surveys and neuropsychological assessment: the Health and Retirement Study and the Aging, Demographics, and Memory Study. *J Gerontol B Psychol Sci Soc Sci*. 2011;66(suppl_1):i162‐i171. doi: 10.1093/geronb/gbr048

10. Langa KM, Plassman BL, Wallace RB, et al. The Aging, Demographics, and Memory Study: study design and methods. *Neuroepidemiology*. 2005;25(4):181–191. doi: 10.1159/000087448

11. Chou R, Dana T, Bougatsos C, Fleming C, Beil T. Screening adults aged 50 years or older for hearing loss: a review of the evidence for the U.S. preventive services task force. *Ann Intern Med*. 2011;154(5):347–355. doi: 10.7326/0003‐4,819‐154‐5‐201,103,010‐00009

12. Nandi A, Glymour MM, Subramanian SV. Association among socioeconomic status, health behaviors, and all‐cause mortality in the United States. *Epidemiology*. 2014;25(2):170–177. doi: 10.1097/EDE.0000000000000038

13. National Institute on Alcohol Abuse and Alcoholism. Drinking levels defined. September 19, 2023. https://www.niaaa.nih.gov/alcohol-health/overview-alcohol-consumption/moderate-binge-drinking

14. Jenkins CD, Stanton BA, Niemcryk SJ, Rose RM. A scale for the estimation of sleep problems in clinical research. *J Clin Epidemiol*. 1988;41(4):313–321. doi: 10.1016/0895‐4,356(88)90138‐2

15. Watson D, Clark LA, Tellegen A. Development and validation of brief measures of positive and negative affect: the PANAS scales. *J Pers Soc Psychol*. 1988;54(6):1063–1,070. doi: 10.1037/0022‐3514.54.6.1063

16. Brim OG, Featherman DL. Surveying midlife development in the United States. Published online 1998. September 19, 2023.

17. Mroczek DK, Kolarz CM. The effect of age on positive and negative affect: a developmental perspective on happiness. *J Pers Soc Psychol*. 1998;75(5):1333–1,349. doi: 10.1037/0022‐3514.75.5.1333

18. Watson D, Clark LA. The PANAS‐X: manual for the positive and negative affect schedule‐expanded form. Published online 1994. September 19, 2023. doi: 10.17077/48vt‐m4t2

19. Diener ED, Emmons RA, Larsen RJ, Griffin S. The Satisfaction With Life Scale. *J Pers Assess*. 1985;49(1):71–75. doi: 10.1207/s15327752jpa4901_13

20. Scheier MF, Carver CS, Bridges MW. Distinguishing optimism from neuroticism (and trait anxiety, self‐mastery, and self‐esteem): a reevaluation of the Life Orientation Test. *J Pers Soc Psychol*. 1994;67(6):1063–1,078. doi: 10.1037/0022‐3514.67.6.1063

21. Ryff CD, Keyes CLM. The structure of psychological well‐being revisited. *J Pers Soc Psychol*. 1995;69(4):719–727. doi: 10.1037/0022‐3514.69.4.719

22. Abbott RA, Ploubidis GB, Huppert FA, Kuh D, Wadsworth ME, Croudace TJ. Psychometric evaluation and predictive validity of Ryff's psychological well‐being items in a UK birth cohort sample of women. *Health Qual Life Out*. 2006;4(1):76. doi: 10.1186/1477‐7,525‐4‐76

23. Lachman ME, Weaver SL. The sense of control as a moderator of social class differences in health and well‐being. *J Pers Soc Psychol*. 1998;74(3):763–773. doi: 10.1037/0022‐3514.74.3.763

24. Radloff LS. The CES‐D Scale: a self‐report depression scale for research in the general population. *Appl Psychol Meas*. 1977;1(3):385–401. doi: 10.1177/014662167700100306

25. Steffeck D. Documentation of affective functioning measures in the Health and Retirement Study. Ann Arbor, MI: University of Michigan. Published 2000. September 19, 2023. https://hrs.isr.umich.edu/sites/default/files/biblio/dr-005.pdf.

26. Beck AT, Weissman A, Lester D, Trexler L. The measurement of pessimism: the Hopelessness Scale. *J Consult Clin Psych*. 1974;42(6):861–865. doi: 10.1037/h0037562

27. Everson SA, Kaplan GA, Goldberg DE, Salonen R, Salonen JT. Hopelessness and 4‐year progression of carotid atherosclerosis: the Kuopio Ischemic Heart Disease Risk Factor Study. *Arterioscler Thromb Vas*. 1997;17(8):1490–1,495. doi: 10.1161/01.atv.17.8.1490

28. Beck AT, Epstein N, Brown G, Steer RA. An inventory for measuring clinical anxiety: psychometric properties. *J Consult Clin Psych*. 1988;56(6):893–897. doi: 10.1037//0022‐006x.56.6.893

29. Wetherell JL, Areán PA. Psychometric evaluation of the Beck Anxiety Inventory with older medical patients. *Psychol Assessment*. 1997;9(2):136–144. doi: 10.1037/1040‐3590.9.2.136

30. Forgays DK, Spielberger CD, Ottaway SA, Forgays DG. Factor structure of the State–Trait Anger Expression Inventory for middle‐aged men and women. *Assessment*. 1998;5(2):141–155. doi: 10.1177/107319119800500205

31. Lee Y, Bierman A. A longitudinal assessment of perceived discrimination and maladaptive expressions of anger among older adults: does subjective social power buffer the association? *J Gerontol B Psychol Sci Soc Sci*. 2018;73(8):e120‐e130. doi: 10.1093/geronb/gbw110

32. Cook WW, Medley DM. Proposed hostility and pharisaic‐virtue scales for the MMPI. *J Appl Psychol*. 1954;38(6):414–418. doi: 10.1037/h0060667

33. Turner RJ, Wheaton B, Lloyd DA. The epidemiology of social stress. *Am Sociol Rev*. 1995;60(1):104–125. doi: 10.2307/2096348

34. Williams DR, Yu Y, Jackson JS, Anderson NB. Racial differences in physical and mental health: socio‐economic status, stress and discrimination. *J Health Psychol*. 1997;2(3):335–351. doi: 10.1177/135910539700200305

35. Essed P. *Understanding Everyday Racism: An Interdisciplinary Theory*. Sage Publications; 1991. doi: 10.4135/9781483345239

36. Feagin JR. The continuing significance of race: antiblack discrimination in public places. *Am Sociol Rev*. 1991;56(1):101–116. doi: 10.2307/2095676

37. Russell DW. UCLA Loneliness Scale (Version 3): reliability, validity, and factor structure. *J Pers Assess*. 1996;66(1):20–40. doi: 10.1207/s15327752jpa6601_2

38. Schuster TL, Kessler RC, Aseltine Jr RH. Supportive interactions, negative interactions, and depressed mood. *Am J Commun Psychol*. 1990;18(3):423–438. doi: 10.1007/BF00938116

39. Smith EN, Romero C, Donovan B, et al. Emotion theories and adolescent well‐being: results of an online intervention. *Emotion*. 2018;18(6):781–788. doi: 10.1037/emo0000379

40. Turner RJ, Frankel BG, Levin DM. Social support: conceptualization, measurement, and implications for mental health. *Res Community Ment Health*. 1983;3:67–111. https://psycnet.apa.org/record/1984‐20538‐001

41. Adler NE. The MacArthur Scale of Subjective Social Status. https://macses.ucsf.edu/research/psychosocial/subjective.php. Published 2007. September 19, 2023.

**TABLE A1:** Changes in the raw score of sense of control from the pre‐baseline wave (t_0_) to the outcome wave (t_2_)*

	Pre‐baseline wave (t_0_)	Outcome wave (t_2_)
	Mean (SD)	Mean (SD)
Sense of control	4.76 (0.97)	4.77 (0.98)

* The correlation coefficient for sense of control between waves t_0_ and t_1_ was 0.41. Further, the correlation coefficient for sense of control between waves t_1_ and t_2_ was 0.32. The numbers in the table are raw scores, and not standardized scores. Further, the actual mean change in sense of control from the Pre‐Baseline Wave (t0) to the Outcome Wave (t2) was −0.0107.

**TABLE A2:** Characteristics of participants at pre‐baseline: retained versus dropped out.

	Retained (*N* = 9960)		Dropped out (*N* = 3811)	
Participant characteristics	No. (%)	Mean (SD)	No. (%)	Mean (SD)
Sense of control		4.84 (0.93)		4.55 (1.03)
Sociodemographic factors				
Age		67.76 (8.89)		733.11 (10.41)
Female (%)	5982 (60.06)		2059 (54.03)	
Race/ethnicity (%)				
White	7668 (77.00)		2.974 (78.04)	
Black	1268 (12.73)		493 (12.94)	
Hispanic	806 (8.09)		282 (7.40)	
Other	217 (2.18)		62 (1.63)	
Married (%)	6433 (64.59)		2156 (56.57)	
Annual household income (%)				
<$50,000	5692 (57.15)		2670 (70.06)	
$50,000–$74,999	1647 (16.54)		480 (12.60)	
$75,000–$99,999	953 (9.57)		223 (5.85)	
>$100,000	1668 (16.75)		438 (11.49)	
Total wealth (%)				
1st quintile	1848 (18.55)		922 (24.19)	
2nd quintile	1934 (19.42)		810 (21.25)	
3rd quintile	1982 (19.90)		767 (20.13)	
4th quintile	1848 (20.70)		695 (18.24)	
5th quintile	2134 (21.43)		617 (16.19)	
Education (%)				
<High school	1726 (17.37)		989 (25.99)	
High school	5484 (55.18)		2029 (53.32)	
>College	2728 (27.45)		787 (20.68)	
Health insurance (%)	9504 (95.47)		3679 (96.61)	
Geographic region (%)				
Northeast	1443 (14.51)		648 (17.04)	
Midwest	2687 (27.02)		907 (23.86)	
South	3952 (39.73)		1545 (40.64)	
West	1864 (18.74)		702 (18.46)	
Personality factors				
Openness (range: 1–4)		2.96 (0.55)		2.85 (0.60)
Conscientiousness (range: 1–4)		3.38 (0.46)		3.27 (0.52)
Extraversion (range: 1–4)		3.22 (0.54)		3.13 (0.56)
Agreeableness (range: 1–4)		3.54 (0.46)		3.49 (0.50)
Neuroticism (range: 1–4)		2.04 (0.61)		2.06 (0.63)
Childhood abuse (%)	643 (6.54)		206 (5.57)	
Health behaviors				
Frequent physical activity (%)	7547 (75.85)		2322 (60.99)	
Smoking (%)	1171 (11.84)		554 (14.65)	
Heavy drinking (%)	593 (7.32)		198 (6.13)	
Sleep problems (%)	2234 (41.14)		821 (44.86)	
Physical health				
Number of physical conditions (range: 0–8)		2.50 (1.40)		2.96 (1.57)
Diabetes (%)	1800 (18.07)		928 (24.35)	
Hypertension (%)	5482 (55.04)		2365 (62.06)	
Stroke (%)	612 (6.14)		495 (12.99)	
Cancer (%)	1325 (13.30)		752 (19.73)	
Heart disease (%)	2045 (20.53)		1309 (34.35)	
Lung disease (%)	734 (7.37)		564 (14.80)	
Arthritis (%)	5824 (58.47)		2467 (64.73)	
Overweight/obese (%)	7108 (72.24)		2383 (63.24)	
Physical functioning limitations (%)	7961 (20.07)		1330 (34.90)	
Cognitive impairment (%)	1564 (15.87)		1141 (30.90)	
Chronic pain (%)	3332 (33.46)		1422 (37.33)	
Self‐rated health (range: 1–5)		3.27 (1.06)		2.83 (1.12)
Hearing (range: 1–5)		3.37 (1.07)		3.17 (1.14)
Eyesight (range: 1–6)		4.24 (0.96)		4.03 (1.05)
Psychological well‐being				
Positive affect (range: 1–5)		3.61 (0.73)		3.48 (0.77)
Life satisfaction (range: 1–7)		5.09 (1.44)		4.87 (1.52)
Optimism (range: 1–6)		4.51 (0.96)		4.32 (0.94)
Purpose in life (range: 1–6)		4.65 (0.91)		4.38 (0.97)
Mastery (range: 1–6)		4.82 (1.08)		4.58 (1.18)
Health mastery (range: 0–10)		7.39 (2.27)		6.80 (2.65)
Financial mastery (range: 0–10)		7.37 (2.57)		7.25 (2.86)
Psychological distress				
Depression (%)	1232 (12.50)		649 (17.58)	
Depressive symptoms (range: 0–8)		1.30 (1.89)		1.67 (2.02)
Hopelessness (range: 1–6)		2.30 (1.26)		2.65 (1.35)
Negative affect (range: 1–5)		1.65 (0.62)		1.77 (0.69)
Perceived constraints (range: 1–6)		2.14 (1.16)		2.47 (1.28)
Anxiety (range: 1–4)		1.54 (0.57)		1.66 (0.63)
Trait anger (range: 1–4)		2.17 (0.67)		2.15 (0.69)
State anger (range: 1–4)		1.49 (0.50)		1.51 (0.54)
Cynical hostility (range: 1–6)		2.93 (1.13)		3.02 (1.15)
Stressful life events (range: 0–5)		0.24 (0.57)		0.19 (0.50)
Financial strain (range: 1–5)		1.97 (1.00)		1.94 (1.00)
Daily discrimination (range: 1–6)		1.63 (0.74)		1.60 (0.76)
Major discrimination (range: 0–6)		0.49 (0.89)		0.40 (0.85)
Social factors				
Loneliness (range: 1–3)		1.47 (0.54)		1.52 (0.55)
Living with spouse/partner (%)	6603 (68.03)		2193 (59.71)	
Contact children (%)				
<Every few months	1260 (12.96)		585 (15.88)	
1–2×/month	1094 (11.25)		416 (11.29)	
1–2×/week	3093 (31.81)		1041 (28.25)	
>3×/week	4276 (43.98)		1643 (44.59)	
Contact other family (%)				
<Every few months	2295 (23.53)		979 (26.59)	
1–2×/month	2332 (23.91)		792 (21.51)	
1–2×/week	2721 (27.90)		958 (26.02)	
>3×/week	2405 (24.66)		953 (25.88)	
Contact friends (%)				
<Every few months	1509 (15.38)		759 (20.51)	
1–2×/month	1816 (18.51)		666 (18.00)	
1–2×/week	3573 (35.42)		1237 (33.42)	
>3×/week	2912 (29.68)		1039 (28.07)	
Closeness with spouse (range: 1–4)		3.47 (0.73)		3.49 (0.76)
Number of close children		2.78 (3.64)		2.90 (3.96)
Number of close other family		3.85 (5.64)		3.94 (5.35)
Number of close friends		4.57 (6.00)		4.46 (6.12)
Positive social support from spouse (range: 1–4)		3.47 (0.64)		3.43 (0.69)
Positive social support from children (range: 1–4)		3.27 (0.72)		3.28 (0.73)
Positive social support from other family (range: 1–4)		2.88 (0.87)		2.91 (0.88)
Positive social support from friends (range: 1–4)		3.06 (0.74)		3.02 (0.76)
Social strain from spouse (range: 1–4)		1.98 (0.67)		1.97 (0.71)
Social strain from children (range: 1–4)		1.71 (0.63)		1.66 (0.65)
Social strain from other family (range: 1–4)		1.57 (0.62)		1.56 (0.62)
Social strain from friends (range: 1–4)		1.85 (0.42)		1.83 (0.43)
Volunteering (%)				
0 h/year	6075 (61.07)		2853 (74.93)	
1–49 h/year	1231 (12.37)		299 (7.85)	
50–99 h/year	868 (8.73)		217 (5.70)	
100–199 h/year	946 (9.51)		245 (6.44)	
>200 h/year	828 (8.32)		193 (5.07)	
Helping friends/neighbors/relatives (%)				
0 h/year	4354 (43.85)		2272 (59.79)	
1–49 h/year	2495 (25.13)		723 (19.03)	
50–99 h/year	1468 (14.78)		368 (9.68)	
100–199 h/year	928 (9.36)		261 (6.87)	
>200 h/year	683 (6.88)		176 (4.63)	
Religious service attendance (%)				
Not at all	2285 (22.95)		1170 (30.74)	
<1×/week	3159 (31.73)		1141 (29.98)	
>1×/week	4512 (45.32)		1495 (39.28)	
Social status ladder (range: 1–10)		6.56 (1.72)		6.39 (1.83)
Change in social status ladder (%)				
Moved down	930 (9.65)		338 (9.58)	
No change	7419 (76.7)		2876 (77.79)	
Moved up	1290 (13.38)		381 (12.63)	
In labor force (%)	3941 (39.57)		841 (22.07)	

*Notes*: This table was created based on non‐imputed data. All variables in Table 1 were used as covariates and assessed in the pre‐baseline wave (*t*_0_; 2006/2008). The percentages in some sections may not add up to 100% due to rounding.

**TABLE A3:** Candidate predictors of mastery (Health and Retirement Study [HRS]: *N* = 13,771).

Candidate predictor	Beta	95% CI
Health behaviors		
Frequent physical activity	0.01	−0.06, 0.07
Smoking	0.01	−0.13, 0.14
Heavy drinking	0.03	−0.10, 0.15
Sleep problems	−0.07	−0.11, −0.02**
Physical health		
Number of physical conditions	−0.03	−0.10, 0.04
Diabetes	−0.04	−0.14, −0.06
Hypertension	0.01	−0.08, 0.09
Stroke	−0.02	−0.10, 0.13
Cancer	0.04	−0.13, 0.05
Heart disease	0.01	−0.10, 0.12
Lung disease	0.04	−0.16, 0.08
Arthritis	−0.05	−0.18, 0.08
Overweight/obese	−0.04	−0.14, 0.06
Physical functioning limitations	−0.09	−0.16, −0.01*
Cognitive impairment	−0.01	−0.10, 0.08
Chronic pain	−0.06	−0.13, 0.01
Self‐rated health	0.07	0.03, 0.11**
Hearing	0.01	−0.02, 0.04
Eyesight	0.04	0.01, 0.07**
Psychological well‐being		
Positive affect	0.13	0.10, 0.16***
Life satisfaction	0.11	0.07, 0.14***
Optimism	0.08	0.05, 0.12***
Purpose in life	0.11	0.07, 0.15***
Health mastery	0.10	0.07, 0.12***
Financial mastery	0.10	0.07, 0.12***
Psychological distress		
Depression	−0.09	−0.17, −0.02*
Depressive symptoms	−0.05	−0.07, −0.02***
Hopelessness	−0.09	−0.12, −0.07***
Negative affect	−0.09	−0.12, −0.05***
Anxiety	−0.07	−0.11, −0.04***
Trait anger	−0.03	−0.06, 0.00
State anger	−0.01	−0.03, 0.02
Cynical hostility	−0.01	−0.04, 0.01
Stressful life events	−0.02	−0.04, 0.00
Financial strain	−0.03	−0.06, 0.00
Daily discrimination	−0.05	−0.07, −0.02***
Major discrimination	−0.01	−0.05, 0.03
Social factors		
Loneliness	−0.06	−0.08, −0.03***
Living with spouse	−0.10	−0.19, −0.01*
Contact children		
<Every few months	Reference	Reference
1–2×/month	0.02	−0.08, 0.13
1–2×/week	0.06	−0.04, 0.15
>3×/week	0.09	0.00, 0.19
Contact other family		
<Every few months	Reference	Reference
1–2×/month	0.03	−0.02, 0.08
1–2×/week	0.03	−0.04, 0.09
>3×/week	0.07	0.02, 0.13*
Contact friends		
<Every few months	Reference	Reference
1–2×/month	0.05	−0.06, 0.16
1–2×/week	0.04	−0.05, 0.13
>3×/week	0.09	0.00, 0.18
Closeness with spouse	−0.01	−0.08, 0.06
Number of close children	0.01	−0.02, 0.05
Number of close other family	0.02	−0.02, 0.05
Number of close friends	0.02	−0.02, 0.06
Positive social support from spouse	0.04	−0.02, 0.10
Positive social support from children	0.06	0.02, 0.10*
Positive social support from other family	0.01	−0.02, 0.05
Positive social support from friends	0.03	−0.01, 0.07
Social strain from spouse	−0.05	−0.13, −0.02
Social strain from children	−0.03	−0.08, 0.02
Social strain from other family	0.00	−0.03, 0.02
Social strain from friends	0.01	−0.02, 0.05
Volunteer		
0 h/year	Reference	Reference
0–49 h/year	0.04	−0.02, 0.10
50–99 h/year	0.00	−0.09, 0.09
100–199 h/year	0.08	−0.00, 0.17
>200 h/year	0.07	−0.03, 0.17
Helping friends/neighbors/relatives		
0 h/year	Reference	Reference
1–49 h/year	0.03	−0.03, 0.09
50–99 h/year	0.02	−0.05, 0.10
100–199 h/year	0.05	−0.05, 0.15
>200 h	0.10	0.00, 0.19*
Religious service attendance		
Not at all	Reference	Reference
<1×/week	0.03	−0.03, 0.10
>1×/week	0.00	−0.06, 0.06
Social status ladder	0.08	0.04, 0.11**
Change in social status ladder		
Moved down	Reference	Reference
No change	0.08	‐0.05, 0.20
Moved up	0.10	‐0.01, 0.20
In labor force	0.09	0.02, 0.15**

*Notes*: The analytic sample was restricted to those who had participated in the pre‐baseline wave (2006 or 2008). Multiple imputation was performed to impute missing data on the exposures, covariates, and outcome. Candidate antecedents were assessed, one at a time, in wave 2 (2010/2012), and the outcome (sense of control) was assessed in wave 3 (2014/2016). The following covariates were controlled for at wave 1 (2006/2008): sociodemographic characteristics (age, sex, race/ethnicity, marital status, income, total wealth, level of education, health insurance, geographic region), personality factors (openness, conscientiousness, extraversion, agreeableness, neuroticism), childhood abuse, and all of the predictor variables, including health behaviors (physical activity, smoking, heavy drinking, sleep problems), physical health (heart disease, cancer, stroke, arthritis, hypertension, overweight/obese, diabetes, lung disease, chronic pain, hearing, eyesight, self‐rated health, physical functioning limitations, cognitive impairment), social factors (living with spouse, frequency of contact with children, frequency of contact with other family, frequency of contact with friends, loneliness, closeness with spouse, number of close children, number of close other family, number of close friends, positive social support from spouse, positive social support from children, positive social support from friends, positive social support from other family, social strain from spouse, social strain from children, social strain from other family, social strain from friends, religious service attendance, volunteering, helping friends/neighbors/relatives, perceived social status, change in perceived social status), psychological well‐being factors (life satisfaction, positive affect, purpose in life, optimism, health mastery, financial mastery), psychological distress (depressive symptoms, hopelessness, negative affect, anxiety, trait anger, state anger, daily discrimination, major discrimination, cynical hostility, stressful life events, financial strain), in labor force), and pre‐baseline values of the outcome (sense of control). All continuous candidate antecedents were standardized (mean = 0; standard deviation = 1). An exposure‐wide analytic approach was used, and a separate model for each exposure was run. Higher scores indicate higher sense of mastery. The *p* value cutoff for Bonferroni correction is *p* = .05/59 predictors = *p* < .00084.

Abbreviations: CI, confidence interval; RR, risk ratio.

* *p* < .05 before Bonferroni correction.

** *p* < .01 before Bonferroni correction.

*** *p* < .05 after Bonferroni correction.

**TABLE A4:** Candidate predictors of perceived constraints (Health and Retirement Study [HRS]: *N* = 13,771).

Candidate predictor	Beta	95% CI
Health behaviors		
Frequent physical activity	−0.01	−0.05, 0.04
Smoking	0.08	−0.03, 0.20
Heavy drinking	0.01	−0.14, 0.17
Sleep problems	0.02	−0.04, 0.08
Physical health		
Number of physical conditions	−0.03	−0.03, 0.09
Diabetes	0.03	−0.06, 0.12
Hypertension	0.02	−0.08, 0.11
Stroke	0.07	−0.06, 0.20
Cancer	−0.08	−0.17, 0.01
Heart disease	0.01	−0.10, 0.13
Lung disease	−0.08	−0.20, 0.04
Arthritis	0.06	0.05, 0.16
Overweight/obese	0.07	0.01, 0.12*
Physical functioning limitations	0.05	−0.02, 0.13
Cognitive impairment	0.04	−0.02, 0.10
Chronic pain	0.02	−0.03, 0.07
Self‐rated health	−0.05	−0.09, −0.02**
Hearing	0.00	−0.04, 0.04
Eyesight	−0.02	−0.05, 0.01
Psychological well‐being		
Positive affect	−0.10	−0.15, −0.06***
Life satisfaction	−0.06	−0.10, −0.03***
Optimism	−0.12	−0.15, −0.09***
Purpose in life	−0.11	−0.16, −0.06***
Health mastery	−0.06	−0.09, −0.03***
Financial mastery	−0.07	−0.11, −0.03**
Psychological distress		
Depression	0.07	−0.04, 0.17
Depressive symptoms	0.04	−0.01, 0.09
Hopelessness	0.14	0.09, 0.18***
Negative affect	0.09	0.05, 0.12***
Anxiety	0.09	0.05, 0.13***
Trait anger	0.04	0.02, 0.06***
State anger	0.04	0.02, 0.07***
Cynical hostility	0.08	0.05, 0.10***
Stressful life events	0.01	−0.03, 0.06
Financial strain	0.05	0.00, 0.09
Daily discrimination	0.05	0.02, 0.08*
Major discrimination	0.01	−0.04, 0.07
Social factors		
Loneliness	0.06	0.03, 0.09***
Living with spouse	0.03	−0.08, 0.14
Contact children		
<Every few months	Reference	Reference
1–2×/month	−0.07	−0.18, 0.05
1–2×/week	−0.05	−0.14, 0.04
>3×/week	−0.05	−0.15, 0.05
Contact other family		
<Every few months	Reference	Reference
1–2×/month	−0.04	−0.08, 0.03
1–2×/week	−0.01	−0.10, 0.08
>3×/week	0.03	−0.07, 0.13
Contact friends		
<Every few months	Reference	Reference
1–2×/month	−0.04	−0.15, 0.08
1–2×/week	−0.06	−0.21, 0.08
>3×/week	−0.05	0.17, 0.07
Closeness with spouse	0.01	−0.05, 0.06
Number of close children	0.00	−0.03, 0.03
Number of close other family	0.01	−0.01, 0.03
Number of close friends	0.00	−0.05, 0.04
Positive social support from spouse	−0.03	−0.07, 0.01
Positive social support from children	−0.04	0.07, 0.01**
Positive social support from other family	0.01	−0.02, 0.05
Positive social support from friends	−0.01	−0.05, 0.03
Social strain from spouse	0.04	0.02, 0.07***
Social strain from children	0.07	0.04, 0.10***
Social strain from other family	0.03	0.00, 0.06*
Social strain from friends	0.00	−0.04, 0.04
Volunteer		
0 h/year	Reference	Reference
0–49 h/year	−0.04	−0.12, 0.04
50–99 h/year	−0.03	−0.10, 0.05
100–199 h/year	−0.07	−0.18, 0.03
>200 h/year	−0.10	−0.19, −0.01*
Helping friends/neighbors/relatives		
0 h/year	Reference	Reference
1–49 h/year	−0.03	−0.08, 0.02
50–99 h/year	−0.03	−0.11, 0.05
100–199 h/year	−0.02	−0.11, 0.07
>200 h	−0.09	−0.19, 0.01
Religious service attendance		
Not at all	Reference	Reference
<1×/week	−0.02	−0.11, 0.06
>1×/week	0.01	−0.07, 0.08
Social status ladder	−0.03	−0.07, 0.00
Change in social status ladder		
Moved down	Reference	Reference
No change	‐0.06	‐0.13, 0.02
Moved up	‐0.09	‐0.20, 0.01
In labor force	‐0.03	‐0.12, 0.06

*Notes*: The analytic sample was restricted to those who had participated in the pre‐baseline wave (2006 or 2008). Multiple imputation was performed to impute missing data on the exposures, covariates, and outcome. Candidate antecedents were assessed, one at a time, in wave 2 (2010/2012), and the outcome (sense of control) was assessed in wave 3 (2014/2016). The following covariates were controlled for at wave 1 (2006/2008): sociodemographic characteristics (age, sex, race/ethnicity, marital status, income, total wealth, level of education, health insurance, geographic region), personality factors (openness, conscientiousness, extraversion, agreeableness, neuroticism), childhood abuse, and all of the predictor variables, including health behaviors (physical activity, smoking, heavy drinking, sleep problems), physical health (heart disease, cancer, stroke, arthritis, hypertension, overweight/obese, diabetes, lung disease, chronic pain, hearing, eyesight, self‐rated health, physical functioning limitations, cognitive impairment), social factors (living with spouse, frequency of contact with children, frequency of contact with other family, frequency of contact with friends, loneliness, closeness with spouse, number of close children, number of close other family, number of close friends, positive social support from spouse, positive social support from children, positive social support from friends, positive social support from other family, social strain from spouse, social strain from children, social strain from other family, social strain from friends, religious service attendance, volunteering, helping friends/neighbors/relatives, perceived social status, change in perceived social status), psychological well‐being factors (life satisfaction, positive affect, purpose in life, optimism, health mastery, financial mastery), psychological distress (depressive symptoms, hopelessness, negative affect, anxiety, trait anger, state anger, daily discrimination, major discrimination, cynical hostility, stressful life events, financial strain), in labor force), and pre‐baseline values of the outcome (sense of control). All continuous candidate antecedents were standardized (mean = 0; standard deviation = 1). An exposure‐wide analytic approach was used, and a separate model for each exposure was run. Higher scores indicate higher sense of perceived constraints. The *p* value cutoff for Bonferroni correction is *p* = .05/59 predictors = *p* < .00084.

Abbreviations: CI, confidence interval; RR, risk ratio.

* *p* < .05 before Bonferroni correction.

** *p* < .01 before Bonferroni correction.

*** *p* < .05 after Bonferroni correction.

**TABLE A5:** Candidate predictors of sense of control: sensitivity analysis with unstandardized variables (Health and Retirement Study [HRS]: *N* = 13,771).

Candidate predictor	Beta	95% CI
Health behaviors		
Frequent physical activity	0.01	−0.04, 0.06
Smoking	−0.05	−0.17, 0.08
Heavy drinking	0.01	−0.09, 0.11
Sleep problems	−0.05	−0.09, −0.01*
Physical health		
Number of physical conditions	−0.02	−0.06, 0.01
Diabetes	−0.04	−0.12, 0.05
Hypertension	0.00	−0.08, 0.07
Stroke	−0.03	−0.11, 0.06
Cancer	0.03	−0.05, 0.11
Heart disease	0.00	−0.08, 0.08
Lung disease	0.02	−0.07, 0.12
Arthritis	−0.06	−0.17, 0.04
Overweight/obese	−0.06	−0.12, 0.00
Physical functioning limitations	−0.07	−0.14, 0.00*
Cognitive impairment	−0.02	−0.09, 0.05
Chronic pain	−0.04	−0.10, 0.01
Self‐rated health	0.07	0.03, 0.10**
Hearing	0.00	−0.02, 0.03
Eyesight	0.03	0.00, 0.06*
Psychological well‐being		
Positive affect	0.17	0.13, 0.21***
Life satisfaction	0.06	0.04, 0.08***
Optimism	0.12	0.09, 0.15***
Purpose in life	0.13	0.08, 0.19***
Health mastery	0.04	0.03, 0.05***
Financial mastery	0.03	0.02, 0.05***
Psychological distress		
Depression	−0.10	−0.18, 0.00
Depressive symptoms	−0.03	−0.04, −0.01**
Hopelessness	−0.10	−0.13, −0.08***
Negative affect	−0.16	−0.19, −0.13***
Anxiety	−0.16	−0.21, −0.11***
Trait anger	−0.06	−0.09, −0.03**
State anger	−0.07	−0.11, −0.03**
Cynical hostility	−0.05	−0.07, −0.03***
Stressful life events	−0.03	−0.08, 0.02
Financial strain	−0.05	−0.08, −0.01*
Daily discrimination	−0.08	−0.11, −0.05***
Major discrimination	−0.01	−0.07, 0.04
Social factors		
Loneliness	−0.13	−0.18, −0.09***
Living with spouse	−0.07	−0.16, 0.01
Contact children		
<Every few months	Reference	Reference
1–2×/month	0.05	−0.04, 0.15
1–2×/week	0.06	−0.03, 0.15
>3×/week	0.09	−0.01, 0.18
Contact other family		
<Every few months	Reference	Reference
1–2×/month	0.03	−0.02, 0.08
1–2×/week	0.02	−0.04, 0.07
>3×/week	0.02	−0.05, 0.09
Contact friends		
<Every few months	Reference	Reference
1–2×/month	0.05	−0.02, 0.12
1–2×/week	0.06	−0.02, 0.13
>3×/week	0.08	0.01, 0.14*
Closeness with spouse	−0.01	−0.06, 0.04
Number of close children	0.00	−0.01, 0.04
Number of close other family	0.00	0.00, 0.00
Number of close friends	0.00	0.00, 0.01
Positive social support from spouse	0.05	−0.01, 0.12
Positive social support from children	0.08	0.04, 0.12***
Positive social support from other family	0.00	−0.02, 0.03
Positive social support from friends	0.03	0.00, 0.07
Social strain from spouse	−0.08	−0.14, −0.02*
Social strain from children	−0.10	−0.15, −0.05***
Social strain from other family	−0.03	−0.08, 0.01
Social strain from friends	0.03	−0.06, 0.10
Volunteer		
0 h/year	Reference	Reference
0–49 h/year	0.05	−0.02, 0.11
50–99 h/year	0.02	−0.06, 0.09
100–199 h/year	0.09	−0.01, 0.18
>200 h/year	0.10	0.01, −0.19*
Helping friends/neighbors/relatives		
0 h/year	Reference	Reference
1–49 h/year	0.04	−0.01, 0.08
50–99 h/year	0.03	−0.03, 0.09
100–199 h/year	0.04	−0.05, 0.13
>200 h	0.11	0.02, 0.19**
Religious service attendance		
Not at all	Reference	Reference
<1×/week	0.03	−0.02, 0.08
>1×/week	0.00	−0.06, 0.05
Social status ladder	0.04	0.01, 0.06**
Change in social status ladder		
Moved down	Reference	Reference
No change	0.08	0.00, 0.15*
Moved up	0.11	0.04, 0.19**
In labor force	0.07	0.01, 0.14*

*Notes*: The analytic sample was restricted to those who had participated in the pre‐baseline wave (2006 or 2008). Multiple imputation was performed to impute missing data on the exposures, covariates, and outcome. Candidate antecedents were assessed, one at a time, in wave 2 (2010/2012), and the outcome (sense of control) was assessed in wave 3 (2014/2016). The following covariates were controlled for at wave 1 (2006/2008): sociodemographic characteristics (age, sex, race/ethnicity, marital status, income, total wealth, level of education, health insurance, geographic region), personality factors (openness, conscientiousness, extraversion, agreeableness, neuroticism), childhood abuse, and all of the predictor variables, including health behaviors (physical activity, smoking, heavy drinking, sleep problems), physical health (heart disease, cancer, stroke, arthritis, hypertension, overweight/obese, diabetes, lung disease, chronic pain, hearing, eyesight, self‐rated health, physical functioning limitations, cognitive impairment), social factors (living with spouse, frequency of contact with children, frequency of contact with other family, frequency of contact with friends, loneliness, closeness with spouse, number of close children, number of close other family, number of close friends, positive social support from spouse, positive social support from children, positive social support from friends, positive social support from other family, social strain from spouse, social strain from children, social strain from other family, social strain from friends, religious service attendance, volunteering, helping friends/neighbors/relatives, perceived social status, change in perceived social status, in labor force), psychological well‐being factors (life satisfaction, positive affect, purpose in life, optimism, health mastery, financial mastery, mastery), psychological distress (depressive symptoms, hopelessness, negative affect, perceived constraints, anxiety, trait anger, state anger, daily discrimination, major discrimination, cynical hostility, stressful life events, financial strain), and pre‐baseline values of the outcome (sense of control). All continuous candidate antecedents were standardized (mean = 0; standard deviation = 1). An exposure‐wide analytic approach was used, and a separate model for each exposure was run. Higher scores indicate higher sense of control. The *p* value cutoff for Bonferroni correction is *p* = .05/59 predictors = *p* < .00084.

Abbreviations: CI, confidence interval; RR, risk ratio.

* *p* < .05 before Bonferroni correction.

** *p* < .01 before Bonferroni correction.

*** *p* < .05 after Bonferroni correction.
